# Protein interactions and consensus clustering analysis uncover insights into herpesvirus virion structure and function relationships

**DOI:** 10.1371/journal.pbio.3000316

**Published:** 2019-06-14

**Authors:** Anna Hernández Durán, Todd M. Greco, Benjamin Vollmer, Ileana M. Cristea, Kay Grünewald, Maya Topf

**Affiliations:** 1 Institute of Structural and Molecular Biology, Birkbeck College, University of London, London, United Kingdom; 2 Division of Structural Biology, Wellcome Centre for Human Genetics, University of Oxford, Oxford, United Kingdom; 3 Department of Molecular Biology, Princeton University, Lewis Thomas Laboratory, Princeton, New Jersey, United States of America; 4 Department of Structural Cell Biology of Viruses, Centre for Structural Systems Biology, Heinrich Pette Institute, Leibnitz Institute of Experimental Virology, University of Hamburg, Hamburg, Germany; University of Glasgow, UNITED KINGDOM

## Abstract

Infections with human herpesviruses are ubiquitous and a public health concern worldwide. Current treatments reduce the severity of some symptoms associated to herpetic infections but neither remove the viral reservoir from the infected host nor protect from the recurrent symptom outbreaks that characterise herpetic infections. The difficulty in therapeutically tackling these viral systems stems in part from their remarkably large proteomes and the complex networks of physical and functional associations that they tailor. This study presents our efforts to unravel the complexity of the interactome of herpes simplex virus type 1 (HSV1), the prototypical herpesvirus species. Inspired by our previous work, we present an improved and more integrative computational pipeline for the protein–protein interaction (PPI) network reconstruction in HSV1, together with a newly developed consensus clustering framework, which allowed us to extend the analysis beyond binary physical interactions and revealed a system-level layout of higher-order functional associations in the virion proteome. Additionally, the analysis provided new functional annotation for the currently undercharacterised protein pUS10. In-depth bioinformatics sequence analysis unravelled structural features in pUS10 reminiscent of those observed in some capsid-associated proteins in tailed bacteriophages, with which herpesviruses are believed to share a common ancestry. Using immunoaffinity purification (IP)–mass spectrometry (MS), we obtained additional support for our bioinformatically predicted interaction between pUS10 and the inner tegument protein pUL37, which binds cytosolic capsids, contributing to initial tegumentation and eventually virion maturation. In summary, this study unveils new, to our knowledge, insights at both the system and molecular levels that can help us better understand the complexity behind herpesvirus infections.

## Abstract 2

Consensus clustering of protein-protein interaction networks provides insights into the assembly mechanism of herpes simplex virus type 1 (HSV1) virions and structure-function relationships underlying herpesvirus infection.

## Introduction

Herpesviruses infect a wide range of eukaryotic organisms and are the etiologic agent of severe diseases in livestock and humans. The nine species of herpesviruses currently known to routinely infect humans are referred to as the human herpesviruses, and their infections are associated with symptoms ranging from fever and cutaneous lesions to encephalitis, meningitis, and a number of cancerous malignancies [1]. A link between herpetic infections and the neurodegenerative Alzheimer’s disease was recently confirmed, emphasising the socioeconomic burden associated with these viruses [2,3].

Herpesviruses are enveloped viruses that assemble into a morphologically unique extracellular particle (i.e., virion), which is organised in concentric structural layers [4]. At the innermost of the virion particle, an icosahedral protein shell of approximately 120 nm diameter—the capsid—encloses the double-stranded DNA (dsDNA) genome of the virus. Surrounding the capsid, a protein matrix—the tegument—occupies about two-thirds of the virion particle volume. The tegument contains proteins that are delivered to the host cytoplasm upon infection and therefore are ready to initiate their function prior to the transcription of any viral genes. The entire particle is coated by a lipid bilayer—the envelope—that contains several proteins and glycoproteins that are crucial for host cell entry and cell-to-cell spread, as well as for modulation of the host’s immune response. As for other enveloped viruses, entry into the host cell occurs via fusion of the envelope with plasma membrane or endocytic cellular membranes, which results in release of the virion content into the host cell cytosol [5]. Altogether, herpesviruses are composed of between approximately 70 to 170 different protein species and achieve diameters between approximately 150 and 250 nm [6,7].

To understand the behaviour of a complex biological system, such as herpes simplex virus type 1 (HSV1), the relationships among the components are as important as the components themselves. The need for a comprehensive description of the relationships among the viral proteins and with host factors has prompted numerous protein–protein interaction (PPI) studies. In 2016, we published a compilation of PPI data in HSV1, the prototypical species of the Herpesviridae family, using integration of experimentally supported data sets from five public resources and computationally predicted PPI data [8]. Taking this work as reference framework, we have now developed an improved computational pipeline for PPI network reconstruction and analysis that is more integrative and statistically robust. In addition to this network reconstruction, the pipeline introduced here incorporates a newly developed consensus clustering protocol for the analysis of the community structure of the resulting network. This protocol was applied to study the community structure in the network formed among proteins present in the HSV1 virion particle. The resulting higher-order relationships predicted previously unrecognised viral protein associations and guided further bioinformatics sequence analysis. Together, the results revealed new, to our knowledge, functional insights at the system and molecular levels. In particular, we focused on the uncovered associations highlighted for the previously undercharacterised Alphaherpesvirinae-specific protein pUS10. We further conducted immunoaffinity purification (IP)–mass spectrometry (MS) experiments in primary human fibroblasts infected with HSV1, which found that proteins pUS10 and protein pUL37 (involved in early tegumentation) specifically coprecipitated, supporting our bioinformatics prediction of a binary interaction between the two proteins.

## Results

### PPI network assembly

#### Data collection from external resources

**Fig 1:**
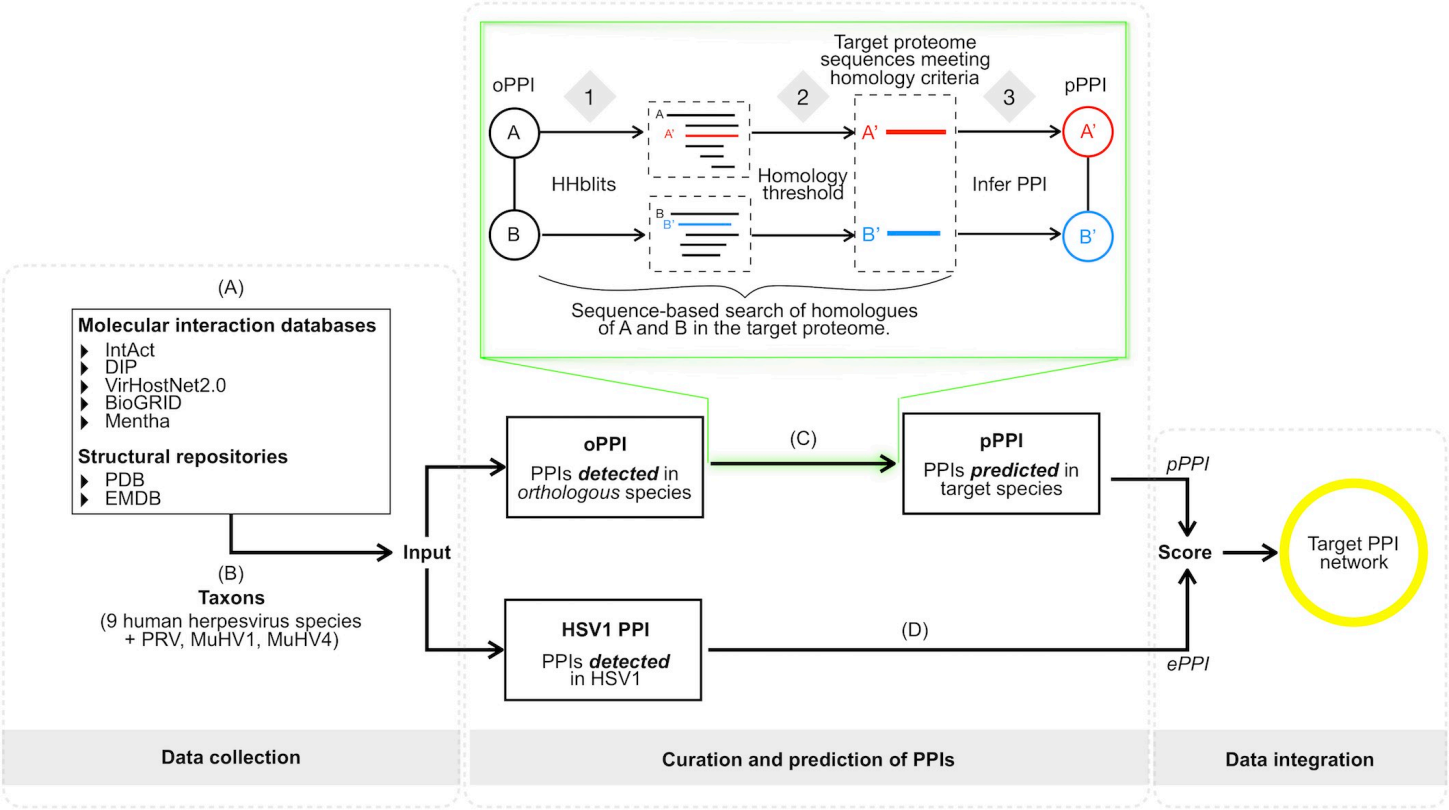
Network assembly framework. (A and B) PPI data for a total of 12 herpesvirus species (nine human and three nonhuman herpesviruses, together covering members of all three subfamilies, i.e., the Alpha-, Beta-, and Gammaherpesvirinae, S1 and S2 Tables) were collected from seven public resources [21]. (C) PPIs detected in any of its orthologous herpesvirus species (oPPIs) were used to predict PPIs in HSV1 (pPPIs). Predictions were conducted based on a sequence-based interologues mapping [22] approach (green box) and included the following steps: for each protein involved in a binary oPPI, (1) sequence-based homologous sequences in the HSV1 proteome were searched for using HHblits [23]; (2) a conservative homology threshold was applied to filter out potential spurious matches among the list of candidates returned by HHblits. From the remaining matches, the best scoring sequence was selected as the most reliable putative HSV1 homologue; (3) if potential HSV1 homologous sequences were found for both proteins in the initial oPPI, an interaction between the two HSV1 sequences was predicted. (D) PPIs experimentally detected in HSV1 were transferred to its interactome (ePPIs). (E) Predicted and experimentally supported PPIs were joined into a nonredundant data set and scored based on their supporting evidence (see Materials and Methods). DIP, Database of Interacting Proteins; EMDB, Electron Microscopy Data Bank; ePPI, experimentally detected PPI; HHblits, HMM-HMM—based lightning-fast iterative sequence search; HSV1, herpes simplex virus type 1; MuHV1, Murid betaherpesvirus; MuHV4, Murid gammaherpesvirus 4; oPPI, PPI in orthologous species; PDB, Protein Data Bank; PPI, protein–protein interaction; pPPI, computationally predicted PPI; PRV, pseudorabies virus.

Binary PPI data were obtained from five molecular interaction repositories (BioGRID [9], the Database of Interacting Proteins [DIP] [10], IntAct [11], Mentha [12], and VirHostNet 2.0 [13]) and two structural databases (Protein Data Bank [PDB] [14] and the Electron Microscopy Data Bank [EMDB] [15]) (Fig 1A). We collected PPIs that have been detected for all nine human herpesvirus species and three closely related nonhuman herpesviruses, i.e., Suid alphaherpesvirus 1 (SuHV1; synonym: pseudorabies virus [PRV]), Murid betaherpesvirus (MuHV1), and Murid gammaherpesvirus 4 (MuHV4), from the Alpha-, Beta-, and Gammaherpesvirinae subfamilies, respectively (Fig 1B and S1 and S2 Tables). The latter were included because these species are frequently used as animal models of herpetic human infections (see Materials and Methods). The resulting joined nonredundant data set contained 2,855 unique pieces of evidence, 2,364 unique PPIs, and 758 unique protein sequences. From this data set, PPIs experimentally detected in species other than HSV1 were used to predict new PPIs in HSV1 (Fig 1C). PPIs experimentally detected in HSV1 were directly added to the network (Fig 1D).

#### Identification of binary PPIs between the portal complex and neighbouring capsid proteins

Our initially curated data set did not contain binary interactions between the portal complex (formed by 12 copies of protein pUL6) and neighbouring capsid proteins. Given the importance of this complex as a capsid component and its demonstrated location in the structure, we conducted a density-fitting analysis using the atomic data on HSV1 capsids published by Dai and Zhou (PDB: 6CGR) [16] and the electron-density map from McElwee (EMBD: 4347) [17].

These data were tentatively located in an initial position in the map (S1 Fig) in such a way that the partial penton would fall in the portal vertex density (because this would be its expected location in the case of a regular vertex). The fit was then adjusted using the Fit-in-map tool in Chimera [18] and with a final cross-correlation value of 0.66. During the fitting, the chain corresponding to the adjacent penton (now falling in the same place as the portal density) was not taken into account because this chain is not represented by the density map. Given the resolution of the density map (7.7 Å), without having the crystal structure of the portal to fit in the map, it is difficult to conclusively determine the exact identity of the physical binary interactions established with neighbouring proteins. However, we combined the observation from McElwee and colleagues [17] with our own results from the current analysis. McElwee and colleagues [17] confidently suggest that the triplex complexes proximal to the portal establish binary contacts with the latter. Our analysis indicates that the most likely candidate for such interactions is pUL18 (virion protein 23 [VP23]). This is in agreement with the expected contacts given the arrangement pattern of the triplexes through the capsid (S1A Fig). By being positioned in a capsid vertex, the portal complex is expected to have the same neighbouring symmetry as the other 11 vertex pentons. Specifically, pentons are flanked by Ta triplexes (one of the six possible orientations of the triplexes). In a Ta orientation, the two copies of pUL18 are most proximal to the penton, and so these are the protein chains most likely to interact with penton subunits. By analogy, pUL18 is also the protein that is most expected to interact with portal complex subunits.

#### Computational prediction of PPIs

To predict PPIs, we used an orthology-based method referred to as ‘interologues mapping’ [19]. This method predicts an interaction in HSV1 if both putative interacting proteins have homologues that are known to interact in other species (see Materials and Methods). Homology relationships are inferred based on sequence-based Hidden Markov Model (HMM) profile alignments [20] in combination with a conservative multicriteria threshold to filter out potential spurious matches from the homology search results (see Materials and Methods).

Experimentally detected interactions in HSV1 and computational predictions were compiled into a PPI network. Because of the large amounts of data integrated during our study, we paid special attention to thoroughly removing duplicated data (S1 Text). Next, the confidence of each interaction based on its cumulative evidence was assessed using a new scoring function (Eqs 1 and 2; see Materials and Methods). The new function is a modification of the MIscore function [24], which was developed by the Proteomics Standards Initiative [25] and is compliant with standardised protocols for the assessment and representation of molecular interaction data [26]. Our scoring function essentially adds a conservative penalty term to the original MIscore function for interactions without experimental support. This term includes information on the prediction method (in this case, sequence-based alignment) and the total number of homologous species from which the prediction was inferred. The function gives higher confidence scores to interactions that have been predicted from a larger number of species. E.g., if an interaction can be predicted in HSV1 based on interactions experimentally detected in three other species of herpesviruses (e.g., varicella-zoster virus [VZV], Epstein–Barr virus [EBV], and human cytomegalovirus [HCMV]), the prediction will score higher than if it had been obtained from a single experimental observation (e.g., only in VZV).

**Fig 2:**
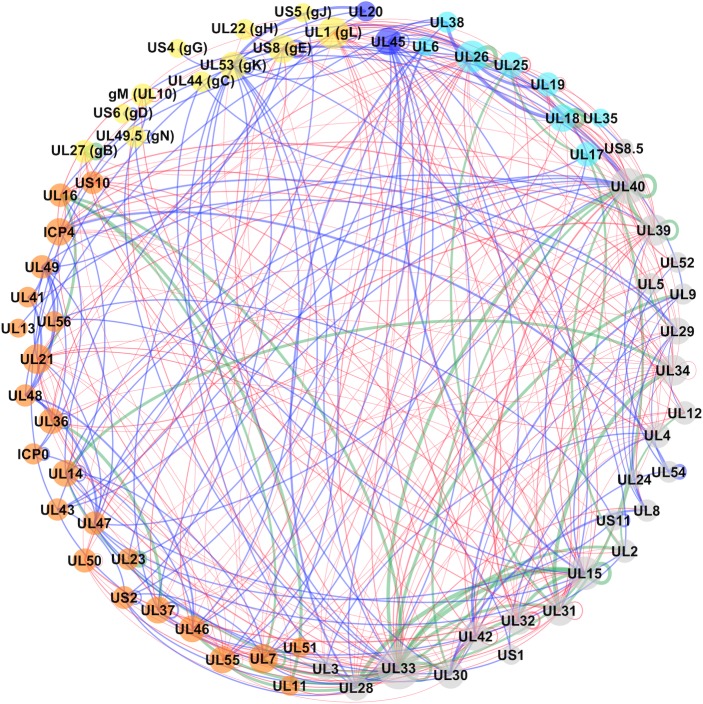
Reconstructed PPI network for HSV1. Nodes represent proteins, and their size is proportional to the number of interactions associated with each protein in the network (degree). Nodes are colour-coded as follows: cyan for capsid and capsid-associated proteins, orange for tegument proteins, yellow for envelope glycoproteins, blue for nonglycosylated envelope proteins, and grey for proteins that are not present in the mature virion particle (i.e., typically only expressed during intracellular stages). Edge (or link) thickness reflects the confidence score for the interaction (the thicker, the higher the confidence). Edges are colour-coded to indicate the type of supporting evidence behind it, i.e., blue for experimentally supported interactions, red for computationally predicted, and green for interactions with both experimental and computational supporting evidence. gB, glycoprotein B; gC, glycoprotein C; gD, glycoprotein D; gE, glycoprotein E; gG, glycoprotein G; gH, glycoprotein H; gK, glycoprotein K; gJ, glycoprotein J; gL, glycoprotein L; gN, glycoprotein N; HSV1, herpes simplex virus type 1; PPI, protein–protein interaction; UL, unique long region; US, unique short region.

The reconstructed network (Fig 2 and S3 Table) contains 370 PPIs formed among 68 proteins and supported by 644 unique pieces of evidence. Among the 370 PPIs, there were 160 experimentally supported interactions (ePPIs) and 250 computationally predicted (pPPIs); 40 interactions were supported by both experimental and computational evidence (ePPIs ∩ pPPIs). All these network data are available through our new version of the HVint database [8] (HVint2.0): http://topf-group.ismb.lon.ac.uk/hvint2/hsv1.html. Five proteins from the HSV1 reference proteome are missing in the current network, i.e., proteins in US12 (ICP47), US3, US9, glycoprotein I (gI or US7), and RL1 (or ICP34.5). The reasons why these proteins are missing are outlined in S2 Text.

### Selection of high-confidence PPI predictions for experimental testing

To try and assess the most confident interactions from our protocol (based on our scoring function), we conducted a manual literature review in search of experimental support for our best predictions (5% top-scoring interactions) among published literature that had not been included in the input data set (i.e., it was not used to infer the predictions). This literature refers specifically to experimental studies that were published prior to our study but not yet integrated in the external databases from which we retrieved our input data. Fully exhaustive curation of published literature is practically not possible at present (even by large curation groups), and hence, it is not surprising to find additional experimental data through manual literature curation. This search returned experimental support for 4 out of the total 11 (top 5% scoring) predicted PPIs examined [8,16,27] (Table 1).

**Table 1:** Top 5% computationally predicted PPIs.

Interacting Protein A	Interacting Protein B	Score
UL32 (P10216)	UL32 (P10216)	0.347
UL32 (P10216)	UL40 (P10224)	0.297
UL5 (P10189)	UL52 (P10236)	0.287
UL25 (P10209)	UL25 (P10209)	0.266
UL15 (P04295)	UL50 (P10234)	0.263
UL40 (P10224)	UL52 (P10236)	0.26
UL39 (P08543)	UL50 (P10234)	0.257
UL23 (P03176)	UL33 (P10217)	0.254
UL21 (P10205)	UL33 (P10217)	0.254
UL5 (P10189)	UL16 (P10200)	0.249
UL12 (P04294)	UL16 (P10200)	0.236

Interacting proteins (A and B) are referred to with their open reading frame name and UniProtKB accession numbers. Shaded rows indicate predicted interactions with additional experimental support that was not included as input during network reconstruction. **Abbreviations:** PPI, protein–protein interaction; UL, unique long region.

### Analysis of the community structure in the HSV1 network

Using the HSV1 network data compiled at this point, we undertook the analysis of the community structure of the subnetwork formed by virion proteins. These are defined as components of the extracellular viral particle (i.e., capsid, tegument, and envelope proteins), which are currently well identified in this species [28–31]. The virion subnetwork on which the clustering protocol was applied contained 44 proteins and 133 edges.

**Fig 3:**
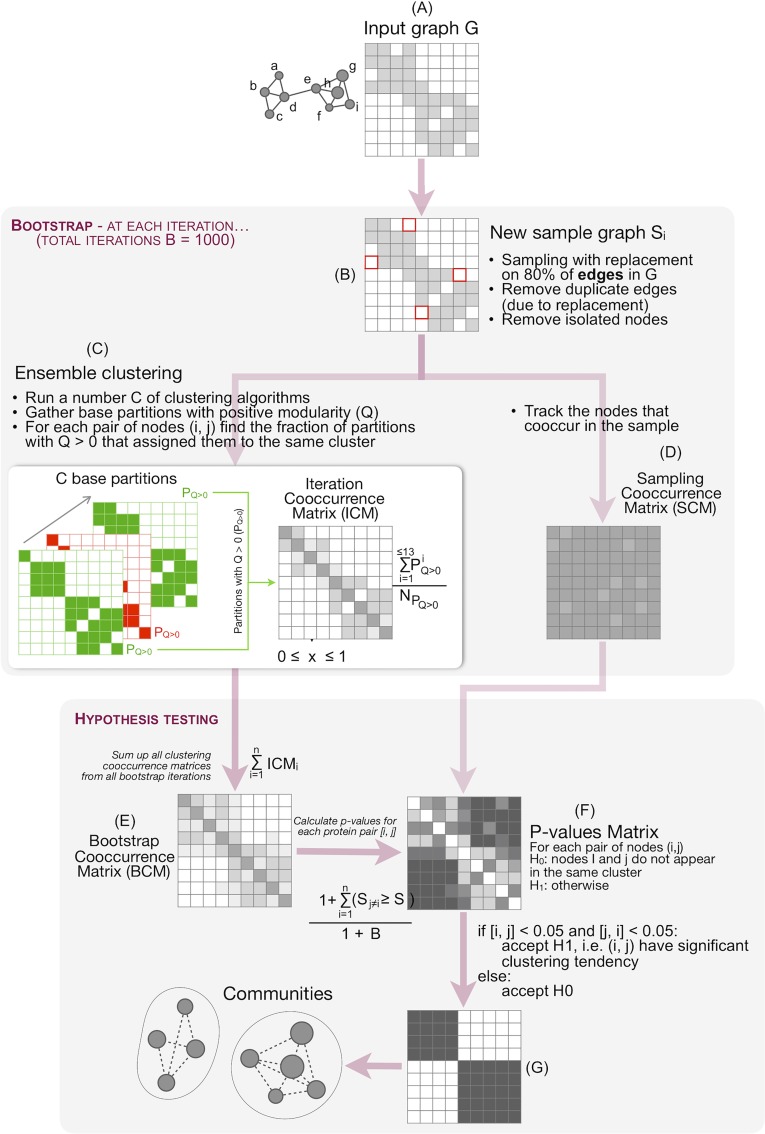
Consensus clustering protocol. (A) Starting from an input network, a series of bootstrap sample graphs are generated. (B) Each sample graph is generated taking 80% of the edges of the input graph *G*. (C) At each bootstrap iteration, 13 clustering algorithms are applied to the sample graph *G*_*i*_. From the resulting partitions, those with positive modularity are integrated into a consensus partition for that iteration (ICM). (D) Throughout the bootstrap procedure, the number of times that two nodes appear in the same sample graph is tracked (SCM) to use later when computing *p*-values. (E) All ICMs are integrated into a BCM. (F) *p*-values are calculated as indicated in Eq 3, using matrices ICM and BCM. (G) Cells with statistically significant values are used to define the final clusters in the network. BCM, bootstrap co-occurrence matrix; ICM, Iteration co-occurrence matrix; SCM, sampling co-occurrence matrix.

Our protocol implements a bootstrap-based clustering consensus approach (see Materials and Methods, Fig 3). At each bootstrap iteration, a new sample graph is created, and 13 different base clustering algorithms are applied to it (Fig 3A–3C; see Materials and Methods). The specific algorithms used were chosen to represent different clustering strategies to reduce the potential intrinsic bias introduced by each approach. Each resulting partition is assessed based on its associated modularity [32]. Only those partitions that show positive modularity are integrated into the iteration consensus clustering partition. Finally, the consensus partitions from all iterations are aggregated into a single bootstrap co-occurrence matrix (BCM) (Fig 3D and 3E). This matrix is used to calculate the statistical significance of the clustering tendency of each pair of nodes *i* and *j* in the network (Fig 3F and 3G). Only those pairs of nodes that satisfied the statistical significance threshold (*p*-value < 0.005) are accepted as members of the same cluster.

**Fig 4:**
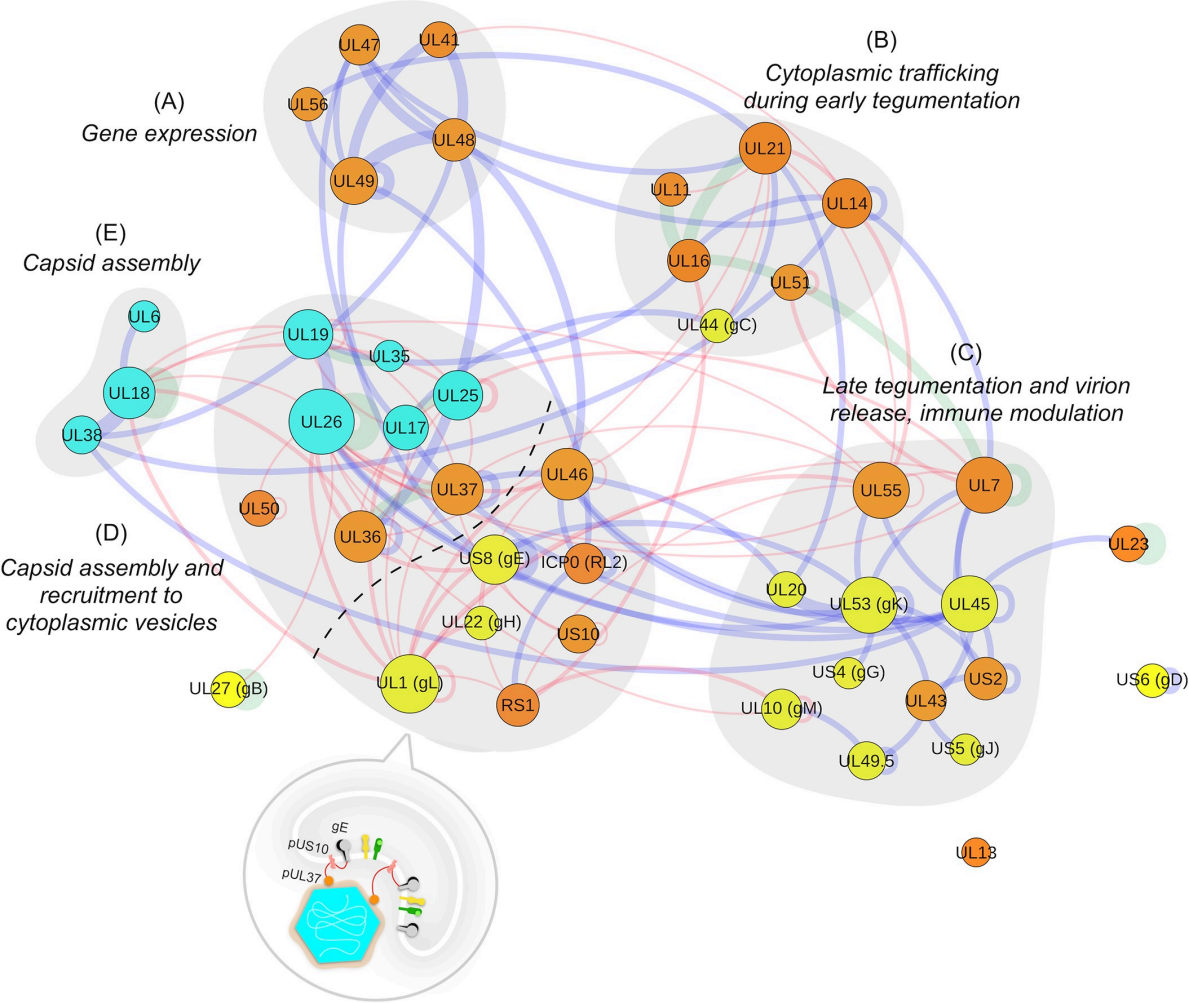
Community structure inferred for the HSV1 virion network. Different communities are delineated by grey areas and labels. Nodes and edges colour-coding follows the same criteria as described in Fig 2, i.e., blue for experimentally supported interactions, red for computationally predicted, and green for interactions with both experimental and computational supporting evidence. The dashed line in community D indicates the two subsets of proteins observed in the community (see text). The associated pictogram reflects the physical and functional relationships among pUS10, pUL37, and gE in the context of the community. gB, glycoprotein B; gC, glycoprotein C; gD, glycoprotein D; gE, glycoprotein E; gG, glycoprotein G; gH, glycoprotein H; gK, glycoprotein K; gJ, glycoprotein J; gL, glycoprotein L; gM, glycoprotein M; HSV1, herpes simplex virus type 1; ICP, infected cell protein; pUL, protein in unique long region; pUS, protein in unique short region; RL, repeat long; RS, repeat short; UL, unique long region; US, unique short region.

The consensus partition divided the network into five communities (Fig 4). Their biological consistency was then assessed using functional annotation data manually curated from gene ontology (GO) [33,34] annotations and published literature (S5 Table).

#### Community A: Gene expression

Community A (proteins pUL41, pUL47, pUL48, pUL49, and pUL56) presents a clear enrichment in proteins related to gene expression modulation. The specific molecular mechanisms by which each of pUL41, pUL47, pUL48, and pUL49 mediate their function differ, yet there is a strong dependence among the four proteins to regulate their activities [35–37]. Additionally, during morphogenesis, pairwise PPIs among these four proteins are required for their incorporation to the tegument [36,38,39]. However, the events that guide the recruitment of this group into the virion are not yet defined. In this context, the fifth protein in the community, i.e., pUL56, is interesting. The latter is a tegument protein previously related to intracellular transport of virion components during tegumentation through interaction with cellular molecular motors and membranes, as well as other virion components involved in tegumentation such as pUL11 [40,41].

#### Community B: Cytoplasmic trafficking during early tegumentation

This community is composed of proteins pUL11, pUL14, pUL16, pUL21, pUL44, and pUL51. The functional interplay among pUL11, pUL16, and pUL21 during early cytoplasmic envelopment is strongly supported (see Discussion) [42–44]. pUL51 is a membrane-anchored protein that has also been shown to be involved in tegumentation, both independently and in complex with other virion components [45,46]. pUL14 has been previously related to intracellular transport of virion components [47]. Importantly, it has been shown to interact with pUL51 [48], and this interaction was not present in the input data set for the reconstruction of the HSV1 interactome. Hence, this provides additional experimental support to the functional relationship suggested by our bioinformatics analysis among this community proteins. The allocation of pUL44 (glycoprotein C or gC) in this community is potentially an artefact of the low number of PPIs between gC in the overall virion subnetwork. However, we cannot discard the functional involvement of gC in virion component trafficking during morphogenesis, as observed for other viral glycoproteins such as gK or glycoprotein E (gE) [49,50]. In this scenario, gC could interact with other community members at, e.g., vesicular membranes.

#### Community C: Late tegumentation and virion release, immune modulation

Community C is defined by proteins involved in late stages of secondary envelopment and virion release. This community is enriched in envelope glycoproteins (gM/gN, gK/UL20, pUL45, pUL43, gJ, and gG) that regulate membrane-associated events. These include internalisation of proteins from the plasma membrane, membrane fusion rates, and modulation of immune responses. Tegument proteins in this community are also annotated with the trafficking of virion components at late stages of virion morphogenesis [51–57].

An interesting observation in community C comes from the presence of pUL55, another poorly characterised α-subfamily–specific protein [58]. Our primary sequence analysis predicted this 186-residue protein to be an α/β protein (S3 Fig, and Materials and Methods). The embedding within this community suggests new functional scenarios in late envelopment and virion release events for the currently poorly understood pUL55 protein.

#### Community D: Capsid assembly and recruitment to cytoplasmic vesicles

Community D holds the most interesting results. This community is enriched in capsid proteins and inner tegument members. Specifically, it contains proteins pUL19 and pUL35, the major and small capsid proteins (MCP and SCP), respectively; pUL26, the capsid scaffolding protein and viral protease; pUL17 and pUL25, members of the heterodimeric capsid-specific vertex component (CSVC); and pUL36 and pUL37, widely accepted components of the inner tegument. The three remaining capsid components (pUL18, pUL38, and pUL6, i.e., the two components of the triplex complex and the portal protein, respectively) were segregated into a small separate community (community E). We believe that these three proteins should be included in the current community D (see Discussion).

Additionally, the community contains a small number of additional proteins. These are proteins ICP0, ICP4, the complex formed by pUL22 (glycoprotein H or gH) and pUL1 (glycoprotein L or gL), pUL46, pUL50, pUS10, and pUS8 (gE). Proteins ICP0, ICP4, and pUL46 are tegument components involved in modulation of transcription. ICP0 and ICP4 are both immediate early (IE) transcription factors with a confirmed but complex functional relationship [59,60]; pUL46 participates in regulating the activity of pUL48 at early stages of infection [35]; pUL50 is the HSV1 deoxyuridine 5′-triphosphate nucleotidohydrolase (dUTPase) [61]. pUL22, pUL1, and pUS8 are envelope glycoproteins. pUL22 and pUL1 form the obligate heterodimer gH/gL known to be involved in viral entry [62–64]; pUS8 is involved in guiding the vesicular trafficking of viral particles and components towards the plasma membranes in coordination with gI and pUS9 in neurons [65,66]. As mentioned earlier, these two proteins (gI and pUS9) were not present in our interactome data set, and that is why they do not appear in the communities (S2 Text).

Taken together, two functionally distinguishable sets of proteins could be identified in this community (one formed by capsid proteins and the other by tegument and envelope proteins in the community). This prompted us to investigate further whether it pointed to potential unknown relationships among these two sets of proteins or to a weakness of our computational pipeline in its ability to discriminate certain types of communities.

Three proteins connect the capsid–inner tegument submodule (pUL19, pUL35, pUL26, pUL17, and pUL25) and the module formed by ICP0, ICP4, pUL46, pUL22, pUL1, pUS8, and pUS10. These connecting proteins are pUL1, pUS8, and pUS10. The association of pUS8 and pUL1 to membranes (through its own transmembrane segment and interaction with the transmembrane protein gH, respectively) has been mentioned above. Protein pUS10 instead is currently a poorly characterised tegument component of the virion particle, specifically found in the Alphaherpesvirinae subfamily [67]. This protein is connected to community members pUS8 and pUL37 through computationally predicted interactions. (see Bioinformatics characterisation of tegument protein pUS10).

The current functional annotation of pUL50, together with the fact that it only presents one single interaction with components of the virion subnetwork (with pUL26), makes us hypothesise that its allocation in this community is not due to real functional associations but rather an artefact of the small number of PPIs with the rest of the network (Fig 4).

### Bioinformatics characterisation of tegument protein pUS10

**Fig 5:**
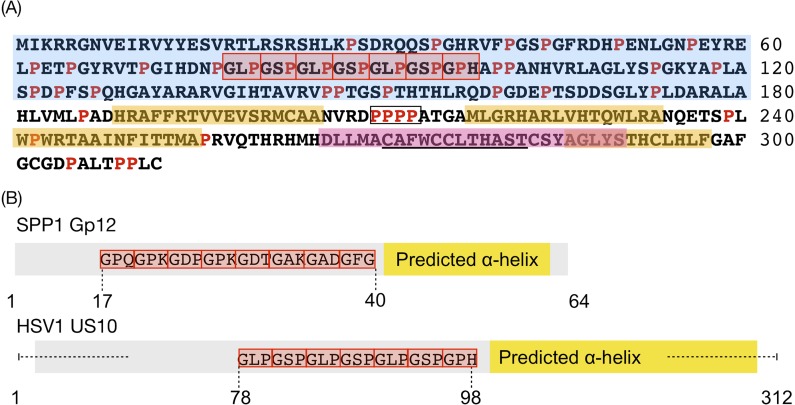
Sequence characterisation of pUS10. (A) Both previously described and newly identified features are indicated. Predicted disordered regions, α-helices, and transmembrane helices are highlighted in blue, yellow, and pink, respectively. CLRs are framed in red boxes. Prolines are highlighted in red. The 4-residue polyproline sequence is framed in a black box. The previously found consensus zinc finger sequence [68] is underscored. (B) Schematic comparison of the structural features of gp12 from bacteriophage SPP1 and pUS10 of HSV1. Protein sequences are shown in grey, CLRs as red boxes with the involved residues annotated, and predicted α-helical regions are highlighted as yellow boxes. CLR, collagen-like repeat; gp12, glycoprotein 12; HSV1, herpes simplex virus type 1; pUS, protein in unique short region; SPP1, secreted phosphoprotein 1.

pUS10 is a poorly characterised Alphaherpesvirinae-subfamily–specific protein of 312 residues that has been found in the nuclear, perinuclear, and cytoplasmic cellular compartments and coprecipitating with capsids, yet direct interactions with capsid proteins have not been reported. It exists in two phosphorylation states and is regarded as a minor component of the tegument [67]. A consensus zinc finger was identified in pUS10 homologues, although the protein failed to bind nucleic acids (common among zinc finger proteins) during experimental testing [67,68]. Finally, HSV1 pUS10 presents a high proline content at its N-terminus and a 4-residue–long polyproline sequence located centrally. To assess whether the clustering of pUS10 with capsid proteins had a functional significance or it was an artefact of a low number of interactions for pUS10 in the input graph, we sought further characterisation of the protein through primary sequence analysis (Fig 5 and S2 Fig; see Materials and Methods). An initial search for potential sequence homologues did not identify any candidates beyond pUS10 counterparts, yet it highlighted the presence of seven tandem collagen-like repeats (CLRs) located towards the N-terminus of the protein sequence. Interestingly, through a manual curation of the literature, we found support for this prediction in a 30-year–old study [69] (note that pUS10 was annotated by its molecular weight (MW) in this study, which could explain why this prediction was not included in databases such as UniProtKB). This prominent feature prompted compelling hypothesis on its functions and evolutionary history (see Discussion). Secondary structure predictions indicated that the N- and C-termini of the protein are structurally distinct. Whilst the N-terminus was predicted to be disordered, the C-terminus was rich in α-helices. Additionally, our predictions revealed a potential single-pass transmembrane segment at the very C-terminus of the protein. This prediction overlaps with the potential zinc finger motif, which could explain why so far, no functional evidence has been provided.

### Experimental support of the pUS10–UL37 interaction by IP–MS

**Fig 6:**
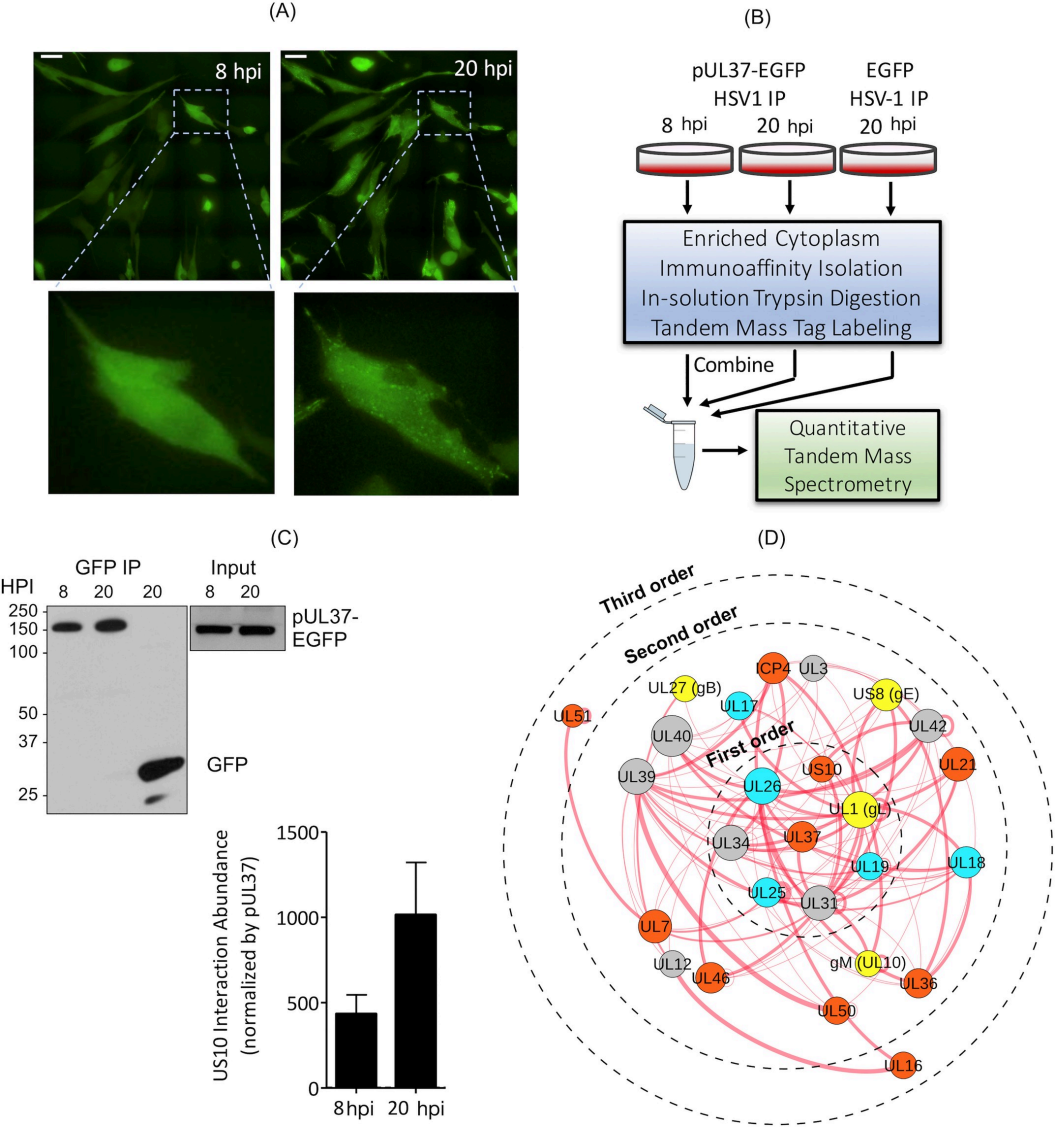
Experimental validation of the pUL37–pUS10 interaction in HSV1-infected primary human fibroblasts. (A) Visualisation of pUL37-EGFP during HSV1 infection of human foreskin fibroblasts using live-cell epifluorescence microscopy. Images show a representative field of infected cells at 8 and 20 HPI. Zoomed images show localisation of pUL37-EGFP (green) in the same cell at 8 and 20 HPI. Scale bar = 50 μm. (B) IP–MS workflow. Human fibroblasts were synchronously infected (multiplicity of infection = 10) with either pUL37-EGFP or EGFP HSV1, with two replicates per condition. HSV1-UL37GFP was collected at 8 and 20 HPI and HSV1-GFP at 20 HPI (HSV1-GFP). pUL37-EGFP and its interactions were isolated from the cytoplasmic cell fraction by IP using anti-GFP antibodies. Proteins were digested with trypsin, and the resulting peptides from each sample were labelled with unique TMT reagents and then combined prior to nanoliquid chromatography–tandem MS analysis. (C) The recovery of pUL37-EGFP and EGFP in the immunoisolates was assessed by western blot with an anti-GFP antibody (S6 Table, columns S-V). 10% of each sample was analysed. The abundance of pUS10 interaction with pUL37 at 8 and 20 HPI (average ± range, *N* = 2). The relative amount of pUS10 was calculated by TMT–MS quantification and normalised by the respective pUL37 TMT abundance in each IP. (D) PPIs around pUL37 present in the reconstructed HSV1 network (Fig 2) and supported by IP results. EGFP, enhanced green fluorescent protein; gB, glycoprotein B; gE, glycoprotein E; GFP, green fluorescent protein; gL, glycoprotein L; gM, glycoprotein M; HPI, hours postinfection; HSV1, herpes simplex virus type 1; ICP, infected cell protein; IP, immunoaffinity purification; MS, mass spectrometry; PPI, protein–protein interaction; pUL, protein in unique long region; pUS, protein in unique short region; TMT, tandem mass tag; UL, unique long region; US, unique short region.

To provide evidence in support of the predicted pUS10–UL37 interaction, we isolated pUL37 from infected cells using IP and analysed the coisolated proteins by quantitative MS (IP–MS). Specifically, protein complexes were isolated from human fibroblasts synchronously infected with HSV1 strains encoding either pUL37 tagged with enhanced green fluorescent protein (pUL37-EGFP) or EGFP alone as a control. Isolations were performed at two functionally distinct time points of infection, 8 and 20 hours postinfection (HPI) (S6 Table). 8 HPI represents an early time point of pUL37 expression, which is prior to secondary envelopment. This is consistent with our observation that pUL37-EGFP appears diffusely localised in the cytoplasm by epifluorescence microscopy (Fig 6A). In contrast, at 20 HPI, secondary envelopment and virus particle release are in progress, reflected by pUL37-EGFP fluorescence visualised as capsid-associated puncta within the cytoplasm and maturing virions (Fig 6A). The observed temporal kinetics and localisation of pUL37 induction were consistent with the prior characterisation of this pUL37-EGFP HSV1 strain [70].

After establishing the temporal expression and cellular distribution of pUL37, we performed IP–MS analyses of pUL37-EGFP and EGFP complexes isolated from cytoplasmic-enriched lysates (Fig 6B). The isolation of pUL37-EGFP from the input lysates was confirmed by western blot (Fig 6C). Next, the coisolated proteins were identified and relatively quantified using labelling with isobaric with tandem mass tags (TMTs). Specifically, proteins were digested with trypsin, and the resulting peptides were derivatised with distinct TMT labels. This approach offered multiplexing, as the labelled pUL37-EGFP and EGFP IPs performed in biological replicates (*N* = 2 replicates) were combined and analysed by tandem MS (Fig 6B). Therefore, both the specificity of the pUL37 interactions (via comparison to the control EGFP IP) and the relative abundance of the interactions at the different time points of infection (8 and 20 HPI) could be simultaneously assessed in this quantitative MS workflow. Nine viral proteins, including pUS10, that appear in our network as having direct interactions with pUL37 were quantitatively enriched by ≥2-fold in pUL37-EGFP versus EGFP control IPs in at least one time point and in both replicate experiments (S6 Table). We observed that the relative amount of pUS10 coisolated with pUL37 was increased at the later stage of infection. At this stage, pUL37-EGFP is localised to sites of secondary envelopment and associates with maturing virions, consistent with the formation of pUL37-EGFP puncta at 20 HPI (Fig 6A). These data suggest a potential role for the pUL37-pUS10 interaction in the cytoplasm during virion maturation.

To more broadly assess the predictions of our computational network with respect to the interaction topology, we examined second- and third-order–interacting partners of pUL37 within our network and compared this subset to our pUL37-EGFP IP–MS results. In our network, 38 proteins had at least one second-order and 16 at least one third-order interaction path to pUL37. Twenty-two of the second-order and eight of the third-order proteins were coisolated with pUL37-EGFP in the IP–MS experiments (see S6 Table). Taken together with the abovementioned direct interactions, 39 out of the 42 proteins coisolated with pUL37-EGFP were also identified as pUL37 protein interactors in our network. Notably, out of the 39 proteins with interaction paths connected to pUL37, 25 were computationally inferred in our network and had no prior experimental support. Given the overlap of these computationally inferred proteins with our pUL37 IP–MS data, these data allowed us to assemble a higher-confidence subnetwork of the 25 proteins computationally predicted to interact with pUL37 (Fig 6D).

## Discussion

Human herpesviruses are ubiquitous human pathogens with a severe socioeconomic impact worldwide [1]. Key to a successful infection is the delicate coordination of the complex network of virus–virus and virus–host protein interactions. Human herpesviruses encode distinctively large proteomes, which translate into large numbers of possible intraviral PPIs. Several studies have attempted to address the description of these networks using a variety of mostly experimental approaches [71–74].

### Key aspects of our PPI network reconstruction protocol

The aim of this study was to undertake a systematic and comprehensive analysis of PPI data in HSV1, the prototypical species of human herpesviruses, to characterise the binary and higher-order relationships among its encoded proteins. To address this, a first step was to design a new computational framework for reconstructing the PPI network. This framework is inspired by our previously published protocol [8] and integrates experimentally supported and computationally predicted binary PPI data in a nonredundant manner using standardised molecular interaction data formats. However, it introduces several key changes (S3 Text). First, it uses the collation of data from a larger number of public resources. Among the added input, our new framework now includes data obtained from high-resolution cryo-electron microscopy, which has progressively placed itself at the forefront of experimental techniques in structural biology. Second, the orthology relationships upon which PPI prediction was based are now established by applying a more stringent set of criteria. Previously, we had used a first-best–hit approach, with no additional requirements (e.g., minimum alignment coverage) to be met by the hits returned from sequence homology searches. Whilst still implementing a first-best–hit approach, we now conservatively filter out potential spurious hits by applying a heuristically derived threshold, which combines information on the alignment coverage, sequence identity and similarity, and probabilistic scores indicating the likelihood of a hit of being a true positive homologue. Third, in this study, we have updated the scaling factor that is used to penalise computationally predicted interactions. The new scaling factor includes information on the prediction method used (i.e., sequence-based orthology) and follows more closely the (nonlinear) mathematical description of the terms in the MIscore function. Additionally, because of the imposition of a more stringent orthology threshold in the current network, as explained above, accounting again for sequence similarity in the scoring function would be redundant. Therefore, here we decided to account for an additional variable, i.e., the degree of conservation across species in the lineage. The reconstructed network is freely available via the newly updated HVint database (HVint2.0) interface: http://topf-group.ismb.lon.ac.uk/hvint2/hsv1.html.

We tested our predictive strategy for the top 5% predicted interactions, and we were able to find independent experimental support (i.e., data not used to build the network) for 4 out of these 11 interactions. These included PPIs between pUL40 and pUL32, pUL40 and pUL52, pUL5 and pUL52, and the homodimer formed by pUL25 [8,16,27]. This highlights the power of our PPI prediction protocol in proposing new interactions for testing.

### Development of a new computational pipeline for network community structure analysis

Our second goal was to learn about higher-order associations among virion proteins and achieve a more thorough understanding of the functional organisation of the underlying binary interactions. Such functional organisation has been shown to be reflected in the structural (topological) arrangement of the corresponding network into communities. In this paper, we have presented the new computational pipeline that we have developed to study the community structure of the HSV1 virion network.

At its core, this pipeline relies on the consensus across a number of base partitions, which are obtained by applying 13 different types of clustering algorithms to the input graph. Partitions, however, can always be created, regardless whether they are meaningful. An extreme case would be, e.g., considering each node as an independent community. We discard such meaningless base partitions from being included in the consensus by imposing them to have positive modularity. This process is embedded in a 1,000-iteration bootstrap procedure. Bootstrap is a statistical technique that allows to calculate the accuracy of an estimate using the available data, and it is particularly useful when the sample size is small [75]. Here, we create a new sample graph at each iteration using 80% of the edges in the original graph. The consensus clustering process described above is then applied to each sample graph. Finally, we use the 1,000 consensus partitions (resulting from the bootstrapping) to measure the *p*-value of the clustering tendency of each pair of proteins. Pairwise associations with *p*-values ≥ 0.005 are discarded, and only those pairwise associations that obtain a *p*-value < 0.005 are further considered. These associations define the final communities.

The community structure resulting from the clustering analysis performed on the virion subnetwork of HSV1 indicates the presence of five large communities (A to E), consistent with distinct stages of the virion formation process (see below). These results suggest that our clustering pipeline is able to identify new and meaningful higher-order functional relationships between proteins.

### Community A

This community sheds light onto early tegumentation stages, specifically on the recruitment of a group of transcription and translation modulators, which play key roles at early stages of the lytic life cycle, into the virion particle. pUL48 (protein in unique long region 48), also called VP16 (virion protein 16) or α-TIF (α-transinducing factor), is a transcription activator that migrates with capsids to the host nucleus upon infection and activates transcription of lytic genes [76]. The roles of pUL47 and pUL49 are more diverse; however, both of them, together with pUL48, participate in regulating the activity of pUL41, also known as the virus host shut-off (VHS) protein, which inhibits protein synthesis [77]. Additionally, binary interactions among the four proteins seem to be required for their incorporation to the virion [35,36,38,78]. Yet, the stage at which this happens and the events that lead the process are unclear. Our results suggest that protein pUL56 could be involved in the recruitment of this module into the particle through a series of coordinated PPIs.

### Communities B and C

Communities B and C are clearly defined by tegumentation events. Community B reflects events that are mostly related to earlier stages of tegumentation, in particular with the migration of capsids from juxtanuclear positions, immediately after nuclear egress of progeny capsids, to other cytoplasmic membranes further away from the nucleus. Specifically, pUL11 is a membrane-anchored protein that localises at sites of secondary envelopment [42,79]; pUL21 has been proposed to participate in intracellular capsid transport through its association to nuclear capsids and, at least in vitro, microtubules [80]; pUL16 has been shown to interact with both pUL11 and pUL21, an interaction that at least in vitro can happen simultaneously [81]. Additionally, pUL16 has also been shown to interact with cytoplasmic capsids [82,83]. Consequently, these three proteins have been suggested to collectively play an active role in the transport of capsids from nuclear and juxtanuclear regions to cytoplasmic membrane envelopment sites through a series of coordinated PPIs [42–44,84]. pUL51 is a membrane-anchored protein that has also been shown to be involved in tegumentation. Although pUL51 is known to interact with pUL7 (in community C), it has also been shown to be able to function independently at earlier stages of virion morphogenesis [45,46]. pUL14 is the least-characterised protein in the cluster; however, its available annotation as involved in intracellular transport of virion components [47] is in agreement with the functional context of the community. Importantly, an interaction with pUL51, which was not present in our input data set, has been experimentally detected [48], providing additional support to the relationship among these two community B members.

Community C, on the other hand, is enriched in proteins that participate at later stages of tegumentation, such as migration from cytoplasmic vesicles to the cellular plasma membrane for virion release. We observed a large number of proteins annotated with trafficking of trans-Golgi network (TGN)-derived vesicles and membrane-regulatory events. A number of proteins also seem to be involved in immune-modulation processes. Our results also provide a new functional context for the currently poorly characterised Alphaherpesvirinae-specific protein pUL55 [58]. Primary sequence analysis indicates pUL55 is a globular α/β protein with no predicted transmembrane regions.

### Communities D and E

The split between D and E as separate communities indicates that, in this particular case, our clustering method had been too stringent. The most likely reason for this is that although there are five interactions between communities D and E, these are all based on computational predictions, whereas the two interactions within community E are experimentally supported and, importantly, highly scoring. Our clustering pipeline uses the information on the confidence scores of each interaction (which characterises the cumulative weight of each edge in the input graph). Therefore, a large difference in the weights within and between communities, respectively, could potentially lead to an artificial splitting of the community.

Nevertheless, the overall functional consistency across the identified communities in the partition gives us confidence that they are biologically informative. Community D also leads to suggesting novel, to our knowledge, functional relationships between inner/midtegument proteins with envelope components that could explain so far uncharacterised steps in capsid tegumentation, specifically, on the recruitment of progeny capsids to cytoplasmic vesicles for their trafficking and sorting to the plasma membrane at late stages of virion morphogenesis. In this context, community D might also be of importance in the formation of so-called L-particles, i.e., capsidless virions, which have been inferred to play an enhancing role in infections by delivering tegument proteins for host manipulation early in infections [85,86].The presence of tegument and envelope proteins ICP0, ICP4, pUL46, pUS10, pUL22, pUL1, and pUS8 in the community, together with the capsid and inner tegument proteins, although surprising at first, triggered our curiosity to further investigate whether these results could be highlighting novel, to our knowledge, functional relationships between the two subgroups. In response to the lack of annotation on protein pUS10, we undertook a bioinformatics analysis of the protein sequence. Additionally, we performed new IP–MS experiments of pUL37 during HSV1 infection to assess the confidence on our computationally predicted interaction between pUS10 and pUL37, which is another central member of the same community.

### Potential relationship of pUS10 to bacteriophages

Protein pUS10 has so far been a poorly characterised minor component of the virion, specific to the Alphaherpesvirinae subfamily. Our primary sequence analysis predicts the sequence to be structurally divided into a disordered N-terminus and an α-helical C-terminus likely to embed a single-pass transmembrane segment. Additionally, our analysis identified seven CLRs centrally in the protein sequence. CLRs are characterised by the GXY pattern, in which X and Y are any amino acid and adopt left-handed helical conformations that tend to tightly pack into trimeric right-handed helices [87,88]. CLRs have been identified in a range of organisms, from multicellular eukaryotes to unicellular bacteria and, to a lesser degree, in viruses, and they participate in a wide range of processes, including, e.g., adhesion, morphogenesis, and regulation of signalling cascades, among others [87–90]. Among herpesviruses, the only other CLR-containing protein that we could identify is the Saimiri transformation-associated protein (STP) from herpesvirus saimiri (HVS), a member of the gamma subfamily. STP is crucial for the transforming (oncogenic) activity of the virus, and it has been reported that the presence of the CLR within the STP sequence is crucial for this activity [91]. However, the presence of a CLR in pUS10 particularly caught our attention, given the overall similarity in secondary structure features and primary sequence motifs that our predictions highlighted with protein glycoprotein 12 (gp12) from the tailed bacteriophage secreted phosphoprotein 1 (SPP1) [92–94]. Seemingly anecdotal, given the overall lack of amino-acid–sequence similarity between the two proteins, a potential link between them is gaining relevance, given the distant but confirmed evolutionary relationships between the tailed bacteriophages and herpesvirus lineages, e.g., reflected in the common existence of a special vertex and a functional portal protein complex [94,95]. Importantly, this evolutionary linkage has been so far established based on structural, functional, and mechanistic similarities rather than sequence similarity. In all but one of the evidences presented so far linking the two lineages, sequence similarity has been, to date, untraceable (S4 Text) [94,96].

It is reasonable to think that new evidence is still to be found. We believe that the observed similarities between pUS10 and gp12 are potentially interesting in this line of investigation and may trigger further investigation. The presence of an envelope in herpesviruses, as opposed to SPP1 virions, could explain the appearance of new features on herpesviral gp12 counterparts, should they exist. One example of such differentiating features could be, e.g., the presence of transmembrane segments, as predicted for pUS10, to assist binding intracellular vesicles during the transit of the capsids across the cytosol in the tegumentation process. In this context, the predicted interaction between pUS10 and gE is especially interesting because gE is known to guide the sorting of capsids and virion components (accumulated in cytoplasmic vesicles) towards the plasma membrane for virion release.

### Experimental validation of the relationship of pUS10 with other community D proteins

Both pUS10 and pUS8 were coisolated with pUL37 in our IP–MS experiments. Importantly, the results of these experiments demonstrate a higher enrichment of pUS10 coisolating with pUL37 later in infection (20 HPI). At these time points, pUL37 localises in secondary envelopment sites [70,97]. The functional role of an interaction between pUS10 and pUL37 could be facilitating the recruitment of the former to sites of secondary envelopment to cooperatively function in subsequent capsid tegumentation events (Fig 4).

Based on the results of these bioinformatics and experimental analyses, together with the functional context drawn from the community, we propose a scenario in which proteins pUS10, pUS8, and pUL37 work in coordination during the recruitment of capsids to cytoplasmic vesicles and participate in the incorporation of tegument components ICP0, ICP4, and pUL46 into the forming virion. Previous experimental studies located ICP0 in the inner tegument layer, whilst ICP4 was found more proximal to the envelope. Importantly, the incorporation of ICP0 and pUS8 in the mature virion has previously been linked through the action of pUL49, the absence of which leads to reduced amounts of both ICP0 and pUS8 proteins in virions [37,98]. On the basis of the structural similarities with gp12, it is reasonable to think that pUS10 could also exhibit reversible binding properties, similar to gp12, and hence could establish dynamic interactions with the capsid or inner tegument proteins whilst, at the same time, interacting with enveloping membranes, e.g., Golgi- or trans-Golgi–derived, through its predicted transmembrane segment. Other interesting features of pUS10 will surely help defining its functionalities. E.g., the polyproline sequence located between two of the predicted C-terminus α-helices is also likely to be involved in PPIs [99].

Altogether, 9 out of the 13 proteins that appear in our network directly interacting with pUL37 were copurified with the latter in our IP experiments (Fig 6D, S5 and S6 Texts). Importantly, these experiments can provide information on higher-order (indirect) interactions, yet not on their topology. Therefore, we decided to couple the experimental data set with second- and third-order interactions to pUL37 obtained from our network. Doing this, we observed a much larger overlap between the two data sets (39 out of the 42 coisolated proteins also present in our pUL37 subnetwork). Hence, these results show a large consistency between our reconstructed network and the newly obtained experimental data, and although the latter cannot conclusively answer which binary interactions are direct or indirect among the coisolated proteins, they add further support to their likely functional association and to the confidence of the reconstructed pUL37-centred subnetwork.

### Summary

In this paper, we have presented a computational pipeline for network reconstruction and community structure analysis. We have applied this pipeline to specifically gain a more thorough characterisation of the relationships among the proteins encoded by the human pathogen HSV1. However, the same analysis could readily be extended to other species. The only prerequisite to implement our network reconstruction framework is the representation of the input molecular interaction data in the standardised format proteomics standards initiative for molecular interactions (PSI-MI) [26]. Similarly, our community structure framework only requires, at present, the input graph to be undirected. We are convinced that our curated PPI data will promote future studies, and with this in mind, we made these data freely available through our new database, HVint2.0 (http://topf-group.ismb.lon.ac.uk/hvint2/hsv1.html).

Collectively, our study brings, to our knowledge, fresh insights, both structural and functional, and at system and molecular levels for the human pathogen HSV1. We note that most tegument proteins in herpesviruses are known to be highly multifunctional, participating in several processes throughout the life cycle. Here, we have elaborated, based on the available functional information, on protein functions that are consistent in the context of the predicted communities. In the case of the currently severely undercharacterised protein pUS10, our predictions open the door to hypothesise novel evolutionary traces with tailed bacteriophages. On balance, our results indicate that our clustering pipeline is able to define functionally consistent and biologically informative communities. We are currently working on the implementation of the introduced network reconstruction and analysis pipelines on other species of human herpesviruses. Widening this analysis to other members of this important group of human pathogens will also underscore both conserved and species-specific features of their interactome organisation and help to explain the observed phenotypes and evolution of their pathogenic strategies.

## Materials and methods

### Data collection, curation, and integration

Binary PPI data from molecular interaction repositories were downloaded in the standardised MITAB 2.5 format [26] (Fig 1). Binary PPIs from PDB and EMDB entries were manually extracted (S7 Table). Only entries with an assigned PubMed identifier were considered. In the case of EMDB entries, only those with resolutions ≤5 Å at the time of this work were taken into account. The only exception made to this criterion was in the case of EMDB entry 4347 (7.7 Å resolution) [17], which was used to extract binary interactions between protein pUL6 and other capsid proteins. As in this case, when an associated atomic model was not available, binary interactions were assigned as described in the primary citation. When atomic models were available, binary interactions between two proteins were extracted if the interface between them was larger than 500 Å^2^ [100–102]. The resulting set of interactions were next manually annotated following the MITAB 2.5 format.

PPI data were exclusively collected for the species in S1 and S2 Tables. Only binary PPIs for which both interacting proteins were annotated with a taxonomic identifier corresponding to these species (all strains considered) were selected. Taxonomic identifiers were obtained from the National Center for Biotechnology Information (NCBI) [103] Taxonomy database (https://www.ncbi.nlm.nih.gov/taxonomy). Once collated, protein identifiers were mapped, where possible, to UniProtKB [7] accession numbers. PPIs for which such mapping was not possible for one or both of the interacting proteins were not further considered in our pipeline. The seven input PPI data sets were merged into a single nonredundant collection.

### Identification of binary PPIs between the portal complex and neighbouring capsid proteins

The atomic model from Dai and Zhou [16] contains a total of 15 chains of the SCP (pUL35), 16 chains of the MCP (pUL19), 5 chains of the triplex protein 1 (pUL38), 10 chains of the triplex protein 2 (pUL18), 1 chain of the CSVC protein pUL17 and 1 of the CSVC protein pUL25, and 1 chain of inner tegument protein pUL36. Together, they represent structural data for 2.5 hexons, 5 neighbouring triplexes (including a Ta triplex proximal to an adjacent penton), 1 chain of such an adjacent penton, and 1 pUL17/pUL25 complex with 1 chain of pUL36.

The atomic map was first segmented using Segger in Chimera [104]. The densities corresponding to the portal complex, and its neighbouring triplexes, hexons, and pUL17/pUL25 + pUL36 complexes (S1 Fig) were selected. The atomic data from Dai and Zhou [16] were then manually positioned into the segmented map. To orient the atomic data on the density map, we allocated the penton atomic data in the portal vertex. The fit was then adjusted using the Fit-in-map tool in Chimera [18].

### Computational prediction of PPIs

PPI predictions were obtained using an interologues mapping approach [19]. This method (Fig 1D) predicts an interaction in species X if two proteins, known to interact in species Y, are conserved in both species X and Y. Here, protein conservation was assessed using sequence-based orthology predictions computed with the iterative HMM profile comparison algorithm implemented by HMM-HMM–based lightning-fast iterative sequence search (HHblits) [23]. For each query sequence, the best match found in HSV1 satisfying all of the following conditions was considered a reliable putative homologue: ≥20% sequence identity, ≥30% sequence similarity, ≥50% query HMM profile coverage, and ≥95% probability of being a true positive. The resulting set of candidate homology relationships were used to infer interologues in each target interactomes.

### Integration of validated and predicted PPIs

Experimentally validated and computationally predicted interactions were merged into a single interactome data set. Strain redundancy was removed by mapping all protein sequences to reference strain accession numbers (HSV1 strain 17) using UniRef90 clusters.

### PPI scoring function

The scoring scheme integrated in our framework is inspired by the standardised MIscore function [24]. Under this scheme, PPIs that had experimental support in the target species (with or without additional support from computational predictions) were scored using the MIscore function. This was done through the MImerge service [24]. PPIs that did not have experimental support were scored with a new scoring function, defined as in Eq 1.

Score=MIscore×(Penaltyfunction) (1)

The new scoring function consists of first scoring an interaction using the MIscore [24] and next applying a penalty function to the returned score. This scaling factor (Eq 2) takes as reference the structure of the terms used in the MIscore function, but it redefines the meaning of their parameters to incorporate information on the number of species and prediction method used.

$$Scalingfactor={log}_{\left(2a+1\right)}(a+1),$$ (2)

where

a=scv×n,

where *scv* is the score associated to the PPI prediction method (in this case, interology mapping) and *n* is the number of different species from which the interaction was predicted.

The value of the penalty function increases asymptotically from approximately 0.5 to 1 with the number of species from which an interaction is predicted. Because we considered 10 orthologous species, the values of the scaling factor in this study fall in the [0.5, 0.6] range. After applying the penalty function, the values are normalised within [0, 1].

### Consensus clustering framework

Our consensus-based clustering strategy was built on a bootstrap process, with *B* = 1,000 the number of bootstrap iterations (Fig 3). Starting from an initial graph *G* (Fig 3A), sample graphs *G*_*i*_ are iteratively generated by selecting 80% of the edges in the original graph *G*. If the obtained *G*_*i*_ contains isolated nodes (i.e., with no links to other nodes in *G*_*i*_), these are removed from *G*_*i*_ (Fig 3B).

For each *G*_*i*_, a total of 13 clustering algorithms were applied (Fig 3C), specifically the K-means algorithm [105], agglomerative hierarchical clustering [106], Fuzzy C-means [107], Model-based clustering [108,109], Markov Cluster algorithm (MCL) [110], Density-based clustering [111], Edge betweenness [32,112], Louvain method [113], Leading eigenvector [114], Fast greedy [115], Walktrap [116], and InfoMap [117] algorithms. Where required, parameter optimisation is guided by either specific metrics commonly associated to a given algorithm or, alternatively, based on modularity maximisation (S5 Table). Because the aim of our study was to delineate nonoverlapping communities, only crisp partitions associated to each of the 13 resulting partitions are taken into account; in the case of fuzzy algorithms, that corresponded to the partition with the highest cluster membership for each node.

Next, partitions with negative or zero modularity are discarded, whilst partitions with positive graph modularity (*P*_*Q* > 0_) are collated and summarised into an Iteration co-occurrence matrix (ICM_*i*_). Each cell in this matrix contains the clustering tendency of a pair of nodes *i* and *j*, as estimated in that particular bootstrap iteration. The clustering tendency of two nodes *i* and *j* is calculated as the fraction of partitions with positive modularity (i.e., *P*_*Q* > 0_) that assigned nodes *i* and *j* in the same cluster (not considering other cluster nodes).

Throughout the bootstrap, we also keep track of the number of bootstrap iterations in which each pair of nodes appears in the simulated graph *G*_*i*_ (sampling co-occurrence matrix [SCM]) (Fig 3D). If a node is not drawn for a given *G*_*i*_, by definition, it will not be clustered with other nodes. This is, however, an artefact of the sampling method and needs to be accounted for to estimate the statistical significance of the clustering tendency for each pair of nodes after the bootstrap procedure. At the end of the bootstrap, all 1,000 ICM_*i*_ are summed up into a BCM (Fig 3E). This is then used to calculate the *p*-value of each pair of nodes (Fig 3F). *P*-values were calculated according to Eq 3.

$$p-value=\frac{1+\sum_{r=0}^{n}{(s}_{r\neq j}\geq s_{j})}{1+B},$$ (3)

where $\sum_{r=0}^{n}{(s}_{r\neq j}\geq s_{j})$ is the total number of cells in row *i* that have a value higher than or equal to the value in cell [*i*, *j*]. *B* is the number of bootstrap iterations. In here, SCM was used to adjust *B* to the exact number of bootstrap co-occurrences for each pair of nodes. The 1 summation on the numerator and denominator is a mathematical correction for a small sample size.

Eq 3 calculates, for each cell [*i*, *j*] in the BCM matrix, the number of cells in row *i* that have a value higher than or equal to cell [*i*, *j*] and scales this value based on the number of bootstrap iterations. Here, we use the SCM to know the exact number of times that two nodes were actually drawn in the same bootstrap iteration (e.g., maybe two nodes were only drawn in the same bootstrap iteration in 995 out of 1,000 of them). Calculated this way, the resulting *p*-values matrix is not symmetric anymore (as opposed to all previously computed matrices) because the significance of cell [*i*, *j*] depends on the clustering tendency profile of *i*, represented by all the values in row *i*, whilst the significance of cell [*j*, *i*] depends on the clustering tendency profile of *j*, i.e., all other cell values in row *j*. Therefore, the statistical significance of [*i*, *j*] and [*j*, *i*], as defined in our study, does not need to be the same. The collection of statistically significant [*i*, *j*] associations derived as here described defines the final clusters in the network (Fig 3G).

### Sequence analysis

The canonical sequence of pUS10 and pUL55 in the HSV1 reference proteome were obtained from UniProtKB [7]. For each sequence, the following analysis was conducted. ScanProsite [118] was used to scan the sequences for sequence motifs. Potential sequence homologues within the entire UniProtKB database [7] were searched for using HHblits [23]. Next, a consensus prediction of secondary structure elements was inferred from the results of four different secondary structure prediction tools, i.e., SPIDER^2^ [119], PSIPRED [120], JPred4 [121], and PSSpred [122]. Probabilities associated to the returned predictions were not integrated in the consensus analysis. Similarly, consensus predictions for transmembrane segments were derived from five different algorithms, i.e., Dense Alignment Surface (DAS) [123], Phobius [124], PHDhtml [125], TMpred [126], and MEMSAT-SVM [127]. From TMpred predictions, only significant regions (defined as regions with score above 500) and core residues were taken into consideration. From Phobius, only residues with probability of belonging to a transmembrane region above 0.1 were considered. Finally, a consensus prediction for disordered regions was built from the results of two algorithms, i.e., DISOPRED [128] and MetaDisorder [129].

### Infection of human fibroblasts with HSV1 and live-cell imaging

We used the HSV1(17^+^)Lox-UL37GFP strain, a generous gift from B. Sodeik and previously characterised in [70], here denoted HSV1-UL37EGFP, and as a control, the HSV1(17^+^)Lox-P_MCMV_GFP strain, denoted HSV1-EGFP, which expresses EGFP alone inserted between the pUL55 and pUL56 ORFs, under the control of the murine cytomegalovirus promoter [130]. Viruses were propagated, isolated, and titred in Vero cells (ATCC CCL81; ATCC, Manassas, VA, USA) grown in DMEM containing 10% FBS and 1% penicillin/streptomycin (P/S), as previously described [8]. Primary human foreskin fibroblast cells were infected with HSV1 strains at 10 plaque-forming units/cell using a cold-synchronised protocol [131]. The progression of infection was visualised by live-cell imaging on a Nikon Ti-Eclipse epifluorescence inverted microscope (Nikon, Tokyo, Japan) from 2 HPI to 24 HPI. Images were viewed and analysed by ImageJ [132].

### IP quantitative MS

For IP–MS experiments, cells were infected as above with HSV1-UL37GFP or control HSV1-GFP, in duplicate. Infected cells were collected at 8 and 20 HPI (HSV1-UL37GFP) or 20 HPI (HSV1-GFP) in ice-cold PBS and pelleted by centrifugation (approximately 1 × 10^7^ per time point per replicate). Cell pellets were washed in ice-cold PBS and lysed hypotonically. Cytosolic lysates were adjusted to 20 mm HEPES-KOH (pH 7.4), containing 0.11 m potassium acetate, 2 mm MgCl_2_, 0.1% Tween 20, 1 μm ZnCl_2_, 1 μm CaCl_2_, 250 mm NaCl, and 0.5% NP-40, mixed by Polytron homogenisation, and centrifuged at 8,000 × *g* for 10 min at 4°C. The supernatant was recovered and subjected to IP using magnetic beads conjugated with in-house generated rabbit anti-GFP antibodies, as previously described [131,133].

Immunoisolated proteins were processed by a Filter-Aided Sample Preparation method using Amicon ultrafiltration devices (30 kDa MWCO; MilliporeSigma, Burlington, MA, USA) as described [20], except 0.1 M Tris-HCl (pH 7.9) was replaced with 0.1 M triethylammonium bicarbonate (TEAB). Following overnight trypsin digestion and clean-up, peptides (4 μl) were analysed by nanoliquid chromatography–tandem MS on a Dionex Ultimate 3000 RSLC coupled directly to an LTQ Orbitrap Velos ETD configured with a Nanospray ion source (Thermo Fisher Scientific, Waltham, MA, USA).

The Proteome Discoverer software (ver. 2.2) was used for postacquisition mass recalibration of precursor and fragment ions masses, MS/MS spectrum extraction, peptide spectrum matching and validation, calculation of TMT reporter ion intensities, and assembly of quantified into protein groups. Protein groups and TMT protein abundances for herpesvirus proteins with a minimum of 2 unique quantified peptides were exported to Excel. IP protein enrichment ratios for each time point and replicate were calculated as the TMT abundance ratio of pUL37GFP/GFP. Proteins with IP enrichment ratios of ≥2-fold in at least one time point in both replicates were considered specific associations. The TMT abundance ratio for proteins in the 20 versus 8 HPI pUL37GFP IPs were calculated after normalisation by the pUL37 TMT abundance. Further details on data collection and analysis can be found in S5 Text.

## Supporting information

S1 Text Redundancy removal. (DOCX)

S2 Text Reference proteome proteins missing in the reconstructed network. (DOCX)

S3 Text Comparison with previous network [8]. (DOCX)

S4 Text Evidence of the evolutionary relationship between Herpesvirales and Caudovirales lineages. (DOCX)

S5 Text Details on IP quantitative MS experiments. IP, immunoaffinity purification; MS, mass spectrometry.
(DOCX)

S6 Text pUL37 subnetwork supported by TMT IP–MS experiments. IP, immunoaffinity purification; MS, mass spectrometry; pUL, protein in unique long region; TMT, tandem mass tag.
(DOCX)

S1 Fig Interactions of the portal complex with neighbouring triplexes. (A) Schematic representation of a capsid vertex to indicate the orientation of Ta triplexes around the penton (in regular vertices) and the portal complex. Yellow and purple hexagons represent the MCP and SCP, respectively; the green pentagon represents a capsid penton, and the triangles represent the heterotrimeric triplexes surrounding the penton. Inside the triangles, the space occupied by the two copies of pUL18 is indicated in red and that occupied by the single copy of pUL38 in red. (B) Density map of the full HSV1 capsid (EMDB: 4347) [17], with the fitted atomic models from PDB: 6CGR [16]. (C) Close-up view of the fitted structures. Here, the map was segmented to show, for clarity, only one hexon, the Ta triplex, and the adjacent pUL17–pUL25 dimer with one pUL36 chain. The heteropentameric CSVC complex that sits on top of the portal complex was capped for visualisation purposes. The map density and the fitted chains are colour-coded as follows. In the density, hexons are coloured in light grey, triplexes in pink, and the portal complex in green. In the fitted structure, the SCP is shown in purple, the MCP in yellow, proteins pUL18 and pUL38 in red and green, respectively, pUL17 in dark blue, pUL25 in magenta, and pUL36 in cyan. CSVC, capsid-specific vertex component; EMDB, Electron Microscopy Data Bank; HSV1, herpes simplex virus type 1; MCP, major capsid protein; PDB, Protein Data Bank; pUL, protein in unique long region; SCP, small capsid protein.
(TIFF)

S2 Fig Primary sequence analysis of pUS10. Previously reported and newly identified features are indicated. Predictions from each software tool are shown. Predicted disordered regions, α-helices, and transmembrane helices are indicated in blue, yellow, and pink, respectively. The identified CLRs are shown in red boxes. Individual prolines are highlighted in red. The 4-residue polyproline sequence is indicated with a black box. The previously identified consensus zinc finger sequence [68] is underscored. The final assignment of the secondary structure elements was based on the consensus of individual methods (prediction confidence scores were not taken into account). CLR, collagen-like repeat; pUS, protein in unique short region.
(TIFF)

S3 Fig Primary sequence analysis of pUL55. Predictions from each software tool are shown. Predicted disordered regions, α-helices, and β-strands are indicated in blue, yellow, and green, respectively. The final assignment of the secondary structure elements was based on the consensus of individual methods (prediction confidence scores were not taken into account). pUL, protein in unique long region.
(TIFF)

S1 Table Herpesvirus species for which PPI data were collected as input for the PPI network assembly framework. PPI, protein–protein interaction.
(XLSX)

S2 Table Taxonomic identifiers associated to species in S1 Table and used to extract PPIs from input resources. (XLSX)

S3 Table PPI network reconstructed for HSV1. For each interaction, the interacting proteins, detection methods, associated PubMed IDs, types of interaction, confidence score, and whether the interaction was computationally predicted and/or experimentally supported are indicated. HSV1, herpes simplex virus type 1; PPI, protein–protein interaction.
(XLSX)

S4 Table Functional annotation for each protein in the reconstructed network. For each protein, the table contains the following information: UniProtKB identifier, ORF name, protein name, presence or absence in the mature virion, and manually curated summaries of cellular and virion location and biological processes in which the protein has been involved (if known). Also given are the sources of this latter annotation. In most cases, this results from a combination of UniProtKB and GO records as well as manually reviewed literature; where appropriate, both PMIDs and the list of GO identifiers associated to the protein are provided. GO, gene ontology; ORF, open reading frame; PMID, PubMed identifier.
(XLSX)

S5 Table Optimisation metrics used on base algorithms. List of metrics used to optimise the base partitions for different algorithms, where required. Popular metrics commonly associated to a given algorithm that are known to give the best performance were prioritised. When such was not available, modularity maximisation was used.
(XLSX)

S6 Table Proteins copurifying with pUL37 by IP–MS. IP, immunoaffinity purification; MS, mass spectrometry; pUL, protein in unique long region.
(XLSX)

S7 Table List of PDB and EMDB entries and associated PubMed ID used as input in our network reconstruction pipeline. In the case of EMDB entries, if fitted PDB structures were present, both IDs are provided. EMDB, Electron Microscopy Data Bank; PDB, Protein Data Bank.
(XLSX)

## Abbreviations

BCM: bootstrap co-occurrence matrix
CLR: collagen-like repeat
CSVC: capsid-specific vertex component
DAS: Dense Alignment Surface
DIP: Database of Interacting Proteins
dsDNA: double-stranded DNA
dUTPase: deoxyuridine 5′-triphosphate nucleotidohydrolase
EBV: Epstein–Barr virus
EGFP: enhanced green fluorescent protein
EMDB: Electron Microscopy Data Bank
ePPI: experimentally detected PPI
gB: glycoprotein B
gC: glycoprotein C
gD: glycoprotein D
gE: glycoprotein E
gH: glycoprotein H
gI: glycoprotein I
gJ: glycoprotein J
gK: glycoprotein K
gL: glycoprotein L
gM: glycoprotein M
gN: glycoprotein N
GO: gene ontology
gp12: glycoprotein 12
HCMV: human cytomegalovirus
HHblits: HMM-HMM–based lightning-fast iterative sequence search
HMM: Hidden Markov Model
HPI: hours postinfection
HSV1: herpes simplex virus type 1
HVS: Herpesvirus saimiri
ICM: Iteration co-occurrence matrix
ICP: infected cell protein
IE: immediate early
IP: immunoaffinity purification
MCL: Markov Cluster algorithm
MCP: major capsid protein
MS: mass spectrometry
MuHV1: Murid betaherpesvirus
MuHV4: Murid gammaherpesvirus 4
MW: molecular weight
NCBI: National Center for Biotechnology Information
PDB: Protein Data Bank
oPPI: PPI in orthologous species
PPI: protein–protein interaction
pPPI: computationally predicted PPI
PSI-MI: proteomics standards initiative molecular interactions
pUL: protein in unique long region
pUS: protein in unique short region
P/S: penicillin/streptomycin
RL: repeat long
RS: repeat short
SCM: sampling co-occurrence matrix
SCP: small capsid protein
SPP1: secreted phosphoprotein 1
STP: Saimiri transformation-associated protein
SuHV1: Suid alphaherpesvirus 1
TEAB: triethylammonium bicarbonate
TGN: trans-Golgi network
TMT: tandem mass tag
UL: unique long region
US: unique short region
VHS: virus host shut-off
VP: virion protein
VZV: varicella-zoster virus
α-TIF: α-transinducing protein
