# Supplementary material for: Protein interactions and consensus clustering analysis uncover insights into herpesvirus virion structure and function relationships
Source: PLoS Biol. 2019 Jun 14;17(6):e3000316. doi: 10.1371/journal.pbio.3000316 (PMC6594648; doi:10.1371/journal.pbio.3000316)
Supplement: S1 Text — (DOCX) [file pbio.3000316.s001.docx]

**S1 Text. Redundancy removal**

Due to the large amount of data integrated during our study, we paid especial attention to thoroughly remove duplicate data. We identified and carefully dealt with the following sources of redundancy:

- Duplication of entries due to overlap across input database entries
- Duplication of entries due to bidirectional annotations of PPIs in different datasets (i.e. A-B and B-A interactions)
- Duplications due to the annotation of the same PPI using different strain identifiers for the proteins involved. For instance, in one database a PPI could be annotated with identifiers HSV1 strain KOS, and in another database it could appear annotated with identifiers from HSV1 strain 17, due to the strains used in the corresponding experiments. Similarly, in the case of predicted PPIs, initial homology mappings can lead to the annotation of PPIs using different strain identifiers. To reduce this type of redundancy, we clustered target sequences in the non-redundant target network using UniRef90 clusters. Subsequently, we selected as cluster representative the sequence corresponding to the reference strain of the target species, i.e. strain 17 for HSV1, strain Merlin for HCMV, and strain B95-8 for EBV. If a cluster did not contain a sequence from the reference strain, the interaction was discarded and not included in the final network.
