# Supplementary material for: Protein interactions and consensus clustering analysis uncover insights into herpesvirus virion structure and function relationships
Source: PLoS Biol. 2019 Jun 14;17(6):e3000316. doi: 10.1371/journal.pbio.3000316 (PMC6594648; doi:10.1371/journal.pbio.3000316)
Supplement: S2 Text — (DOCX) [file pbio.3000316.s002.docx]

**S2 Text. Reference proteome proteins missing in the reconstructed network.**

Five proteins present in the UniProtKB reference proteome of HSV1 are missing in the reconstructed network. These are proteins pUS12 (ICP47), pUS3, pUS9, pUS7 (glycoprotein I or gI), and RL1 (or ICP34.5). pUS3 and RL1 are both multifunctional neurovirulence factors [1-4]. pUS3 is a protein kinase that has been suggested to be involved in multiple processes, by phosphorylating a range of substrates [3,5,6]. RL1 has been found to be implicated in a range of processes including nuclear lamin disruption during capsid egress, and downmodulation of the host immune response by inhibition of autophagy in neurons, interference with the interferon response and expression of MHC class II proteins, as well as with the DNA sensing pathway [2,4,7,8]. Similarly, protein ICP47 inhibits the host immune response by interfering with antigen presentation, ultimately reducing the recognition of infected cells by cytotoxic T cells [9]. Both gI and pUS9 are envelope proteins that work in coordination with pUS8 (glycoprotein E or gE) in sorting viral particles and components; gI does so by forming a permanent and physical complex with gE (gE/gI), whilst pUS9 participates (not as a complex) exclusively in neurons [10].

We inspected on a protein-specific manner, whether our input dataset downloaded from external databases contained any interaction for these five proteins that we could have missed during the implementation of our protocol. We could not identify any interaction in our dataset involving proteins, pUS12, pUS3, and RL1. The dataset contained, however, interactions involving pUS9 from VZV, and for gI from VZV and SuHV1, which we could have presumably computationally map to the HSV1 interactome. In all of these cases though, the results from the sequence homology searches did not provide candidate mappings satisfying the imposed homology criteria, and therefore these interactions had to be discarded. We could find one single interaction involving gI (and pUS1) and detected in HSV1. However, in this record gI was annotated with UniProtKB accession number Q702W3, which was missing in the retrieved UniRef90 clusters, and as a result the interaction was missed in the final network. In the specific case of gI, we were surprised not to find it in our dataset as there exist a PDB entry (PDB ID: 2GJ7) [11], entitled ”Crystal structure of a gE-gI/Fc complex”, and so we expected to find evidence for the complex in these data. Yet, the entry only contains four chains, two of them from gE and the other two from immunoglobulin γ-1 chain C region. Therefore, the entry was not used as evidence of the gE/gI complex.

**References**

1. Michael K, Klupp BG, Karger A, Mettenleiter TC. Efficient incorporation of tegument proteins pUL46, pUL49, and pUS3 into pseudorabies virus particles depends on the presence of pUL21. J Virol. 2007;81: 1048–1051. doi:10.1128/JVI.01801-06

2. Orvedahl A, Alexander D, Tallóczy Z, Sun Q, Wei Y, Zhang W, et al. HSV-1 ICP34.5 confers neurovirulence by targeting the Beclin 1 autophagy protein. Cell Host Microbe. 2007;1: 23–35. doi:10.1016/j.chom.2006.12.001

3. Kato A, Liu Z, Minowa A, Imai T, Tanaka M, Sugimoto K, et al. Herpes simplex virus 1 protein kinase Us3 and major tegument protein UL47 reciprocally regulate their subcellular localization in infected cells. J Virol. 2011;85: 9599–9613. doi:10.1128/JVI.00845-11

4. Manivanh R, Mehrbach J, Knipe DM, Leib DA. Role of Herpes Simplex Virus 1 γ34.5 in the Regulation of IRF3 Signaling. Longnecker RM, editor. J Virol. 2017;91: 30. doi:10.1128/JVI.01156-17

5. Ryckman BJ, Roller RJ. Herpes simplex virus type 1 primary envelopment: UL34 protein modification and the US3-UL34 catalytic relationship. J Virol. American Society for Microbiology (ASM); 2004;78: 399–412. doi:10.1128/JVI.78.1.399-412.2004

6. Mou F, Forest T, Baines JD. US3 of herpes simplex virus type 1 encodes a promiscuous protein kinase that phosphorylates and alters localization of lamin A/C in infected cells. J Virol. American Society for Microbiology; 2007;81: 6459–6470. doi:10.1128/JVI.00380-07

7. Wu S, Pan S, Zhang L, Baines J, Roller R, Ames J, et al. Herpes Simplex Virus 1 Induces Phosphorylation and Reorganization of Lamin A/C through the γ134.5 Protein That Facilitates Nuclear Egress. Longnecker RM, editor. J Virol. American Society for Microbiology; 2016;90: 10414–10422. doi:10.1128/JVI.01392-16

8. Pan S, Liu X, Ma Y, Cao Y, He B. Herpes Simplex Virus 1 γ134.5 Protein Inhibits STING Activation That Restricts Viral Replication. Longnecker RM, editor. J Virol. 2018;92: 1513. doi:10.1128/JVI.01015-18

9. Aubert M, Krantz EM, Jerome KR. Herpes simplex virus genes Us3, Us5, and Us12 differentially regulate cytotoxic T lymphocyte-induced cytotoxicity. Viral Immunol. Mary Ann Liebert, Inc. 2 Madison Avenue Larchmont, NY 10538 USA; 2006;19: 391–408. doi:10.1089/vim.2006.19.391

10. DuRaine G, Wisner TW, Howard P, Williams M, Johnson DC. Herpes Simplex Virus gE/gI and US9 Promote both Envelopment and Sorting of Virus Particles in the Cytoplasm of Neurons, Two Processes That Precede Anterograde Transport in Axons. Longnecker RM, editor. J Virol. American Society for Microbiology Journals; 2017;91: 153. doi:10.1128/JVI.00050-17

11. Sprague ER, Wang C, Baker D, Bjorkman PJ. Crystal structure of the HSV-1 Fc receptor bound to Fc reveals a mechanism for antibody bipolar bridging. Virgin S, editor. PLOS Biology. Public Library of Science; 2006;4: e148. doi:10.1371/journal.pbio.0040148
