# Supplementary material for: Protein interactions and consensus clustering analysis uncover insights into herpesvirus virion structure and function relationships
Source: PLoS Biol. 2019 Jun 14;17(6):e3000316. doi: 10.1371/journal.pbio.3000316 (PMC6594648; doi:10.1371/journal.pbio.3000316)
Supplement: S3 Text — (DOCX) [file pbio.3000316.s003.docx]

**S3 Text. Comparison with previous network (Ashford et al. *Mol Cell Proteomics* 2016)**

The old dataset and the current dataset were compared using the original ids associated to them in the input datasets (i.e. the datasets collated from public databases), which had been kept to map each interaction in the networks with their original evidence source (either computational or experimental).

The current and former datasets contain 370 and 419 PPIs, respectively. The overlap between both datasets is of 287 interactions (over two thirds of each dataset). Therefore, there are 132 and 84 PPIs unique to the former and current datasets, respectively. To understand these differences, we have investigated the reasons why these 132 PPIs present in the former network are missing in the current one.

Among the 132 PPIs unique to the former dataset, 33 had experimental support and 101 had been computationally predicted (pPPI), with 2 interactions both computationally and experimentally supported (tPPI+pPPI). Out the 33 PPIs with experimental support, 25 were missing from the input datasets downloaded from the external databases, which might result from updates in the latter; for 4 other PPIs the identifier of either one or both proteins involved in the interactions was missing among the UniRef90 clusters retrieved from UniProtKB, and therefore the interaction could not be mapped to the reference strain; in the case of 2 other PPIs, our sequence homology searches suggested an alternative best homologous candidate for either or both proteins and that led to a different PPI mapping; finally, for the 2 remaining PPIs, the current dataset did not contain experimental supporting evidence (i.e. they appeared as pPPI candidates only), again possibly due to updates in the external PPIs databases. However, our sequence homology searches did not find any match in HSV1 for either or both of the proteins involved in the original interaction, and so these interactions were discarded.

Similarly, out of the 101 pPPIs predicted in the former network and missing in the current one, 86 of the interactions were discarded because, for either or both proteins involved in the original PPIs, the homology mappings to HSV1 proteome failed to meet one or more of the homology thresholds imposed the current study; 10 PPIs were missing from the initially downloaded input datasets; 2 could not be mapped to the reference proteome due to missing strain identifiers in the UniRef90 clusters; 2 had mapped to alternative PPIs; and for 1 interaction our sequence homology searches failed to find matches with HSV1 proteins for either or both of the proteins involved in the original interaction.
