# Supplementary material for: Protein interactions and consensus clustering analysis uncover insights into herpesvirus virion structure and function relationships
Source: PLoS Biol. 2019 Jun 14;17(6):e3000316. doi: 10.1371/journal.pbio.3000316 (PMC6594648; doi:10.1371/journal.pbio.3000316)
Supplement: S4 Text — (DOCX) [file pbio.3000316.s004.docx]

**S4 Text. Evidence of the evolutionary relationship between Herpesvirales and Caudovirales lineages.**

The evolutionary linkage between the two viral lineages of *Herpesvirales* and Caudovirales (tailed bacteriophages) has so far been established based on structural, functional, and mechanistic similarities, rather than sequence similarity [1]. The strongest evidence of the relationship between the two lineages came from their capsid assembly pathways. Both lineages assemble an icosahedral capsid enclosing the viral dsDNA genome, from an immature form called the procapsid [2,3]. To create the procapsid both herpesviruses and bacteriophages use a helper protein termed scaffolding protein, which is later released from the mature capsid once its formation is completed. Variations exists regarding the number of scaffolding proteins used and whether these are encoded by an independent ORF (as in the case of herpesviruses and most bacteriophages), or constitute a segment of a major capsid protein sequence (as in the case of bacteriophage HK97) [4,5]. Albeit differences in shape (e.g. spherical vs elongated) and symmetry (i.e. T number) occur among bacteriophages, the mature capsid is in both lineages icosahedral, and its faces are composed of true pentameric and hexameric complexes (i.e. pentons and hexons, respectively). These are primarily formed by the major capsid protein (MCP), which holds another striking evidence of a shared common ancestor [1]. First identified in bacteriophage HK97 [6], herpesvirus and tailed bacteriophages MCPs hold a common fold, either comprising the full length or a domain of the protein, which locates at the floor of the capsid, suggesting a key role in the structural transformations occurred during capsid maturation. This MCP fold is distinctive as it is unique in the virosphere (i.e. not observed in other viral lineages to date) [1]. Another capsid component that links the two lineages is the dodecameric portal-complex located in one of the capsid vertices and through which the DNA is injected to and released from the capsid [7,8]. During genome packaging (i.e. insertion into the capsid), a virally-encoded complex, the tripartite terminase, is required to bind the portal complex whilst the capsid is still in its procapsid form, and genome packaging occurs concomitantly to capsid maturation. A tripartite terminase complex is employed by both herpesviruses and tailed bacteriophages, and its large subunit constitutes the only example of sequence conservation between the two lineages found to date [5]. Finally, both tailed bacteriophages and herpesvirus encode auxiliary proteins, which assist the release of the viral genome from the capsid. For this to happen, the genome has to traverse the portal complex and the respective channels at the prokaryotic cell wall and eukaryotic cell nucleus, respectively, prior genome release, triggering the injection across the portal complex and into the cell and nucleus, respectively, to start [5].

**References**

1. Baker ML, Jiang W, Rixon FJ, Chiu W. Common ancestry of herpesviruses and tailed DNA bacteriophages. J Virol. American Society for Microbiology Journals; 2005;79: 14967–14970. doi:10.1128/JVI.79.23.14967-14970.2005

2. Newcomb WW, Homa FL, Thomsen DR, Trus BL, Cheng N, Steven A, et al. Assembly of the herpes simplex virus procapsid from purified components and identification of small complexes containing the major capsid and scaffolding proteins. J Virol. American Society for Microbiology (ASM); 1999;73: 4239–4250.

3. Gertsman I, Gan L, Guttman M, Lee K, Speir JA, Duda RL, et al. An unexpected twist in viral capsid maturation. Nature. 2009;458: 646–650. doi:10.1038/nature07686

4. Cheng H, Shen N, Pei J, Grishin NV. Double-stranded DNA bacteriophage prohead protease is homologous to herpesvirus protease. Protein Sci. 2004;13: 2260–2269. doi:10.1110/ps.04726004

5. Rixon FJ, Schmid MF. Structural similarities in DNA packaging and delivery apparatuses in Herpesvirus and dsDNA bacteriophages. Curr Opin Virol. 2014;5: 105–110. doi:10.1016/j.coviro.2014.02.003

6. Wikoff WR, Liljas L, Duda RL, Tsuruta H, Hendrix RW, Johnson JE. Topologically linked protein rings in the bacteriophage HK97 capsid. Science. 2000;289: 2129–2133.

7. Olia AS, Prevelige PE, Johnson JE, Cingolani G. Three-dimensional structure of a viral genome-delivery portal vertex. Nat Struct Mol Biol. Nature Publishing Group; 2011;18: 597–603. doi:10.1038/nsmb.2023

8. McElwee M, Vijayakrishnan S, Rixon F, Bhella D. Structure of the herpes simplex virus portal-vertex. Sugden B, editor. PLOS Biology. Public Library of Science; 2018;16: e2006191. doi:10.1371/journal.pbio.2006191
