# Supplementary material for: Protein interactions and consensus clustering analysis uncover insights into herpesvirus virion structure and function relationships
Source: PLoS Biol. 2019 Jun 14;17(6):e3000316. doi: 10.1371/journal.pbio.3000316 (PMC6594648; doi:10.1371/journal.pbio.3000316)
Supplement: S5 Text — IP, immunoaffinity purification; MS, mass spectrometry. (DOCX) [file pbio.3000316.s005.docx]

**S5 Text. Details on Immunoaffinity Purification Quantitative Mass Spectrometry experiments.**

We used the HSV1(17^+^)Lox-UL37GFP strain as previously characterized [1], here denoted HSV1-UL37GFP, and as a control, the HSV1(17^+^)Lox-P_MCMV_GFP strain, denoted HSV1-GFP, which expresses EGFP alone inserted between the pUL55 and pUL56 ORFs, under the control of the murine cytomegalovirus promoter [2]. Viruses were propagated, isolated, and titered in Vero cells (ATCC CCL81) grown in DMEM containing 10% FBS and 1% penicillin/streptomycin (P/S), as previously described [3]. Primary human foreskin fibroblast cells were infected with HSV-1 strains at 10 plaque forming units/cell using a cold-synchronized protocol [4]. Briefly, cells were incubated with virus for 1 hr at 4°C in CO_2_-independent DMEM, supplemented with 2% FBS and 0.1% (w/v) BSA. Culture medium was aspirated, and cells were incubated at 37 °C for 1 hr with fresh DMEM supplemented with 2% FBS and 1% P/S. Cells were washed rapidly with citrate buffer (40 mm citrate, 135 mm NaCl, 10 mm KCl, pH 3.0), then replenished with DMEM supplemented with 2% FBS and 1% P/S. The progression of infection was visualized by live-cell imaging on a Nikon Ti-Eclipse epifluorescent inverted microscope from 2 h post infection (hpi) to 24 hpi. Images were viewed and analyzed by ImageJ.

For IP-MS experiments, cells were infected as above with HSV1-UL37GFP or control HSV1-GFP, in duplicate. Infected cells were collected at 8 and 20 hpi (HSV1-UL37GFP) or 20 hpi (HSV1-GFP) in ice-cold PBS and pelleted by centrifugation (~1 x 10^7^ per time point per replicate). Cell pellets were washed in ice-cold PBS and hypotonic lysis was used (10 min on wet ice in 10 mM HEPES-KOH, pH 7.4, containing 1.5 mM MgCl_2_, protease inhibitor cocktail (Sigma Aldrich), and 0.5% NP-40) to obtain a cytosol-enriched fraction. Cytosolic lysates were adjusted to 20 mm HEPES-KOH, pH 7.4, containing 0.11 m potassium acetate, 2 mm MgCl_2_, 0.1% Tween 20, 1 μm ZnCl_2_, 1 μm CaCl_2_, 250 mm NaCl, and 0.5% NP-40, mixed by Polytron homogenization, and centrifuged at 8,000 x g for 10 min at 4°C. The supernatant was recovered and subjected to immunoaffinity purification using magnetic beads conjugated with in-house generated rabbit anti-GFP antibodies, as previously described [4,5].

After immunoisolation, an aliquot of captured proteins (5%) was analyzed by western blot (anti-GFP monoclonal antibody, 1:2000, Roche Diagnostics) using ECL detection (anti-mouse IgG-HRP, 1:5000, Jackson ImmunoResearch) to verify bait protein capture. The remainder of the sample was processed by a Filter-Aided Sample Preparation method using Amicon ultrafiltration devices (Millipore, 30 kDa MWCO) as described [6], except 0.1 M Tris-HCl, pH 7.9 was replaced with 0.1 M triethylammonium bicarbonate (TEAB). Proteins were digested in-filter with 5 ng/µL trypsin in 0.05 M TEAB (100 µL) at 37°C overnight. Digests were recovered by centrifugation and evaporated to ~25 µL by vacuum centrifugation. Samples (GFP @ 20 hpi, pUL37GFP @ 8 hpi, pUL37GFP @ 20 hpi) were treated with a unique isobaric TMT reagent (~0.4 mg in 20 µL of ethanol) for 1 hr at RT with occasional vortexing, followed by addition of 2 µL of 10% hydroxylamine for 20 min to quench excess reagent. Samples within each replicate were combined, concentrated to ~5 µL by vacuum centrifugation, suspended in 0.5% trifluoroacetic acid/5% acetonitrile (ACN) (~50 µL), desalted over C_18_ StageTips [7], concentrated to near dryness, then resuspended in 10 μl of 0.1% formic acid (FA)/5% ACN.

Peptides (4 μl) were analyzed by nanoliquid chromatography-tandem mass spectrometry on a Dionex Ultimate 3000 RSLC coupled directly to a LTQ Orbitrap Velos ETD configured with a Nanospray ion source (Thermofisher Scientific). Briefly, peptides were bound to a trap column (75 μm × 2 cm, Acclaim PepMap100 C18, 3 μm) at 5 μL/min with 0.1% trifluoroacetic acid/1% ACN in water, then eluted and separated by reverse phase chromatography on the analytical column (Acclaim PepMap RSLC, 1.8 μm × 75 μm x 50 cm) at a flow rate of 250 nL/min over a 180 min ACN gradient from 4–35% mobile phase B (A, 0.1% FA in water; B, 98% ACN/0.1% FA in water). Peptides were ionized directly into the mass spectrometer, configured for data-dependent acquisition, with each acquisition cycle comprising a single full-scan mass spectrum (m/z = 385 – 1700) in the orbitrap (r = 30,000 at m/z = 400) followed by HCD MS/MS (fixed first m/z = 100) of the top 10 most intense ions (minimum signal = 2000). Dynamic exclusion was enabled (90 secs) and FT preview scan was disabled.

The Proteome Discoverer software (ver. 2.2) was used for post-acquisition mass recalibration of precursor and fragment ions masses, MS/MS spectrum extraction, and peptide spectrum matching and validation. Mass-recalibrated MS/MS spectra were independently searched by SequestHT (ver 1.17) against the forward and reverse sequences of the human subset of the UniProt-SwissProt database (2016–04), appended with herpesvirus sequences and common contaminants (22,269 sequences). SequestHT search parameters were defined as follows: full trypsin specificity, maximum of 2 missed trypsin cleavages, ion precursor mass tolerance of 5 ppm, fragment ion mass tolerance of 0.02 Da, fixed modification of cysteine carbamidomethylation and TMT at lysine and peptide N-termini, variable modifications of methionine oxidation, asparagine deamidation, and serine, threonine, and tyrosine phosphorylation. TMT reporter ion intensities were extracted from MS/MS spectra using the Reporter Ions Quantifier node with an integration tolerance of 10 ppm using the most confident centroid method. Using peptide spectrum matches to the reverse database, posterior error probabilities and q-values were calculated for each replicate experiment. Replicates were combined in the Proteome Discoverer Consensus workflow and error rates were controlled at the peptide sequence and protein level to 1% FDR. For each peptide, TMT reporter ion signal-to-noise values (average ≥ 6) were corrected for known isotope impurities. TMT protein abundances were calculated as the summation of peptide S/N values that were derived from precursors with co-isolation ≤ 30% and unique to the protein group. Protein groups and TMT protein abundances for herpesvirus proteins with a minimum of 2 unique quantified peptides were exported to Excel. IP protein enrichment ratios for each time point and replicate were calculated as the TMT abundance ratio of pUL37GFP / GFP. Proteins with IP enrichment ratios of ≥ 2-fold in at least one-time point in both replicates were considered specific associations. The TMT abundance ratio for proteins in the 20 vs 8 hpi pUL37GFP IPs were calculated after normalization by the pUL37 TMT abundance.

**References**

1. Sandbaumhüter M, Döhner K, Schipke J, Binz A, Pohlmann A, Sodeik B, et al. Cytosolic herpes simplex virus capsids not only require binding inner tegument protein pUL36 but also pUL37 for active transport prior to secondary envelopment. Cell Microbiol. Wiley/Blackwell (10.1111); 2013;15: 248–269. doi:10.1111/cmi.12075

2. Snijder B, Sacher R, Rämö P, Liberali P, Mench K, Wolfrum N, et al. Single-cell analysis of population context advances RNAi screening at multiple levels. Mol Syst Biol. EMBO Press; 2012;8: 579. doi:10.1038/msb.2012.9

3. Ashford P, Hernandez A, Greco TM, Buch A, Sodeik B, Cristea IM, et al. HVint: A Strategy for Identifying Novel Protein-Protein Interactions in Herpes Simplex Virus Type 1. Mol Cell Proteomics. American Society for Biochemistry and Molecular Biology; 2016;15: 2939–2953. doi:10.1074/mcp.M116.058552

4. Lin AE, Greco TM, Döhner K, Sodeik B, Cristea IM. A proteomic perspective of inbuilt viral protein regulation: pUL46 tegument protein is targeted for degradation by ICP0 during herpes simplex virus type 1 infection. Mol Cell Proteomics. 2013;12: 3237–3252. doi:10.1074/mcp.M113.030866

5. Cristea IM, Williams R, Chait BT, Rout MP. Fluorescent proteins as proteomic probes. Mol Cell Proteomics. American Society for Biochemistry and Molecular Biology; 2005;4: 1933–1941. doi:10.1074/mcp.M500227-MCP200

6. Wiśniewski JR, Zougman A, Nagaraj N, Mann M. Universal sample preparation method for proteome analysis. Nat Methods. Nature Publishing Group; 2009;6: 359–362. doi:10.1038/nmeth.1322

7. Rappsilber J, Mann M, Ishihama Y. Protocol for micro-purification, enrichment, pre-fractionation and storage of peptides for proteomics using StageTips. Nat Protoc. Nature Publishing Group; 2007;2: 1896–1906. doi:10.1038/nprot.2007.261
