# Supplementary material for: Protein interactions and consensus clustering analysis uncover insights into herpesvirus virion structure and function relationships
Source: PLoS Biol. 2019 Jun 14;17(6):e3000316. doi: 10.1371/journal.pbio.3000316 (PMC6594648; doi:10.1371/journal.pbio.3000316)
Supplement: S6 Text — IP, immunoaffinity purification; MS, mass spectrometry; pUL, protein in unique long region; TMT, tandem mass tag. (DOCX) [file pbio.3000316.s006.docx]

**S6 Text. pUL37 subnetwork supported by TMT IP-MS experiments.**

TMT-IP experiments have the ability to provide information not only on binary (direct) interactions but also higher order interactions (indirect), as complexes formed by more than one protein can co-precipitate together. However, these experimental data cannot, by themselves, discriminate the topology of the interactions among the co-isolated proteins. We thus used the data from our reconstructed network (S3 Table) to perform a more in-depth comparison between the pUL37 PPIs identified from the IP-MS to the predicted HSV1 interactions, specifically focusing on higher-order (second and third) interacting neighbours of pUL37.

The specific implementation of the performed experiments cannot identify self-interactions, and therefore these are disregarded in the following analysis. It is also worth emphasising that the analysis described below specifically focuses on the overlap between the experimental results and the subset of predicted interactions in our network for pUL37 (i.e. not interactions that already had experimental support).

Using the list of binary interactions in Table S3, we first identified a total of 38 proteins that established at least one second order interaction to pUL37 (i.e. proteins interacting with direct neighbours of pUL37). Similarly, we identified 16 additional proteins that established third order interactions to pUL37. Then we compared these second and third order datasets to our TMT-IP results. In this context, 22 proteins in the IP were matched to the second order predicted set, while 8 were matched to the third order predicted set.  Taken together, these different degrees (first, second, and third) of predicted interactions correspond to 39 out of 42 proteins in the IP-MS dataset (with specific enrichment in at least one single time point). Note, that we did not include UL24 in this count since it did not meet the specificity criteria. Table S6 includes this information (column E), using numbers 1, 2, and 3 to refer to first (direct), second, third order interactions. Finally, out of these 39 proteins, 25 of them present interaction paths leading to pUL37, which were exclusively computationally predicted in our network (i.e. had no previous experimental support). This subset of 25 proteins formed a predicted subnetwork around pUL37 (Fig 6D) composed of a total of 92 interactions, with 9, 80, and 3 first, second, and third order predicted PPIs to pUL37. This analysis increases the consistency between our reconstructed network and the obtained experimental results and provides additional support to the former.

It is worth noting that we cannot accurately estimate false positive rates of the predicted networks using IP-MS data because the latter cannot be assumed to be a true positive set with 100% sensitivity. In herpesvirus infections in particular, the cascade of viral gene expression and protein translation during infection result in PPI networks also under strict temporal regulation (e.g. Moorman et al. *Mol Cell Proteomics* (2010) [1]). Therefore, we cannot assign (in a statistical sense) a negative interaction based on the absence of that interaction at a particular time point of infection.

**References**

1. Moorman NJ, Sharon-Friling R, Shenk T, Cristea IM. A targeted spatial-temporal proteomics approach implicates multiple cellular trafficking pathways in human cytomegalovirus virion maturation. Mol Cell Proteomics. 2010;9: 851–860. doi:10.1074/mcp.M900485-MCP200
