# Supplementary figures and images for: Protein interactions and consensus clustering analysis uncover insights into herpesvirus virion structure and function relationships
Source: PLoS Biol. 2019 Jun 14;17(6):e3000316. doi: 10.1371/journal.pbio.3000316 (PMC6594648; doi:10.1371/journal.pbio.3000316)

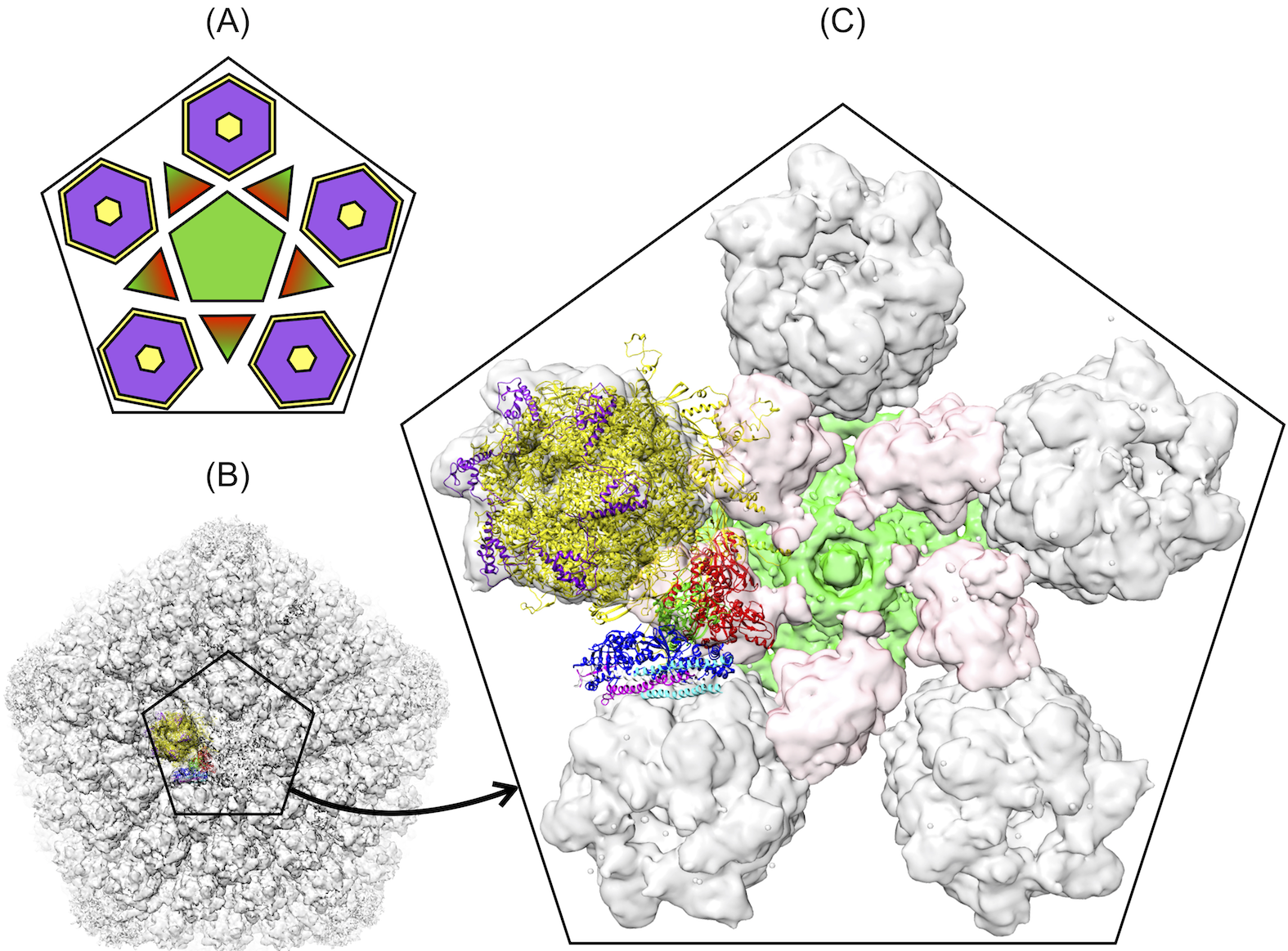

Supplement: S1 Fig — (A) Schematic representation of a capsid vertex to indicate the orientation of Ta triplexes around the penton (in regular vertices) and the portal complex. Yellow and purple hexagons represent the MCP and SCP, respectively; the green pentagon represents a capsid penton, and the triangles represent the heterotrimeric triplexes surrounding the penton. Inside the triangles, the space occupied by the two copies of pUL18 is indicated in red and that occupied by the single copy of pUL38 in red. (B) Density map of the full HSV1 capsid (EMDB: 4347) [17], with the fitted atomic models from PDB: 6CGR [16]. (C) Close-up view of the fitted structures. Here, the map was segmented to show, for clarity, only one hexon, the Ta triplex, and the adjacent pUL17–pUL25 dimer with one pUL36 chain. The heteropentameric CSVC complex that sits on top of the portal complex was capped for visualisation purposes. The map density and the fitted chains are colour-coded as follows. In the density, hexons are coloured in light grey, triplexes in pink, and the portal complex in green. In the fitted structure, the SCP is shown in purple, the MCP in yellow, proteins pUL18 and pUL38 in red and green, respectively, pUL17 in dark blue, pUL25 in magenta, and pUL36 in cyan. CSVC, capsid-specific vertex component; EMDB, Electron Microscopy Data Bank; HSV1, herpes simplex virus type 1; MCP, major capsid protein; PDB, Protein Data Bank; pUL, protein in unique long region; SCP, small capsid protein. (TIFF) [file pbio.3000316.s007.tiff]

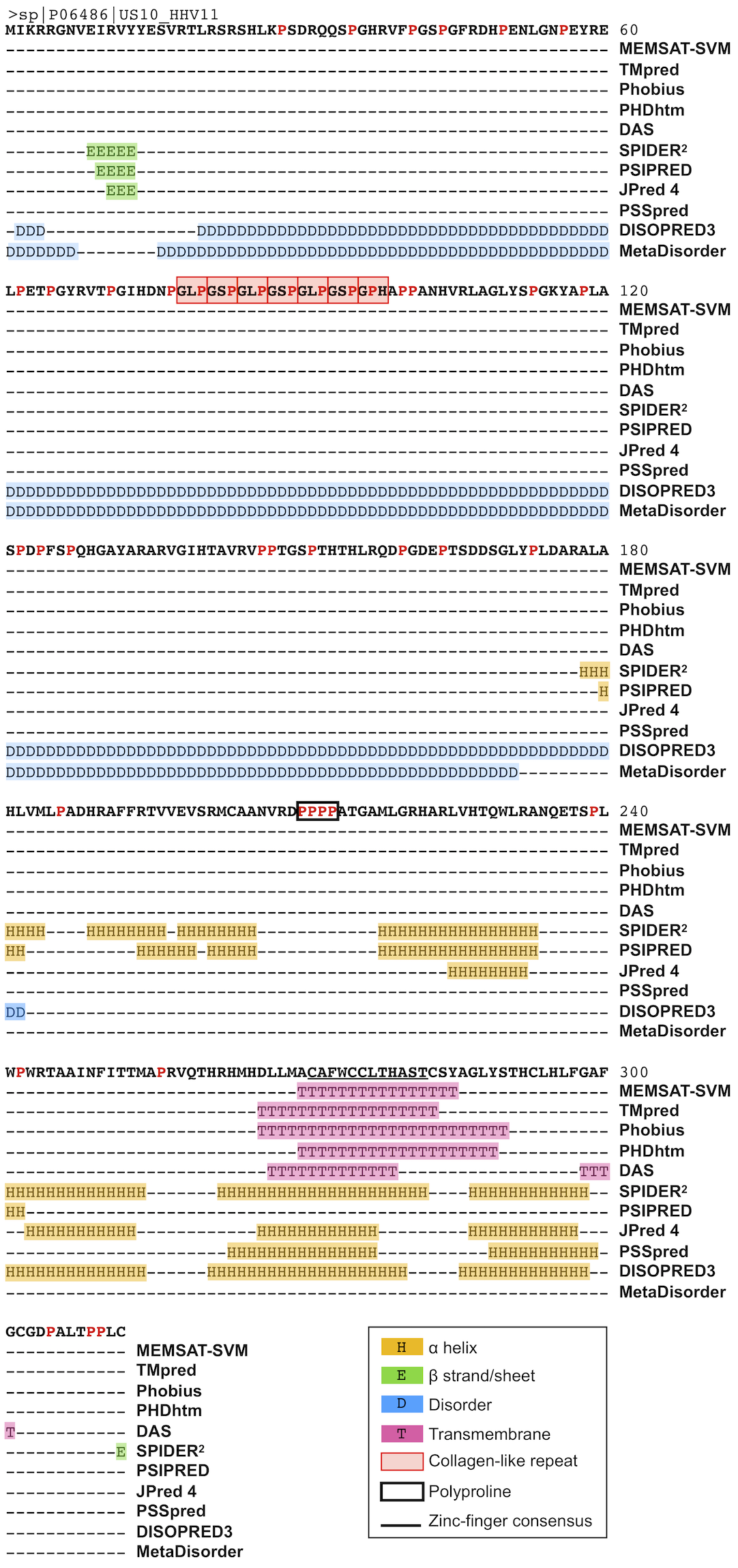

Supplement: S2 Fig — Previously reported and newly identified features are indicated. Predictions from each software tool are shown. Predicted disordered regions, α-helices, and transmembrane helices are indicated in blue, yellow, and pink, respectively. The identified CLRs are shown in red boxes. Individual prolines are highlighted in red. The 4-residue polyproline sequence is indicated with a black box. The previously identified consensus zinc finger sequence [68] is underscored. The final assignment of the secondary structure elements was based on the consensus of individual methods (prediction confidence scores were not taken into account). CLR, collagen-like repeat; pUS, protein in unique short region. (TIFF) [file pbio.3000316.s008.tiff]

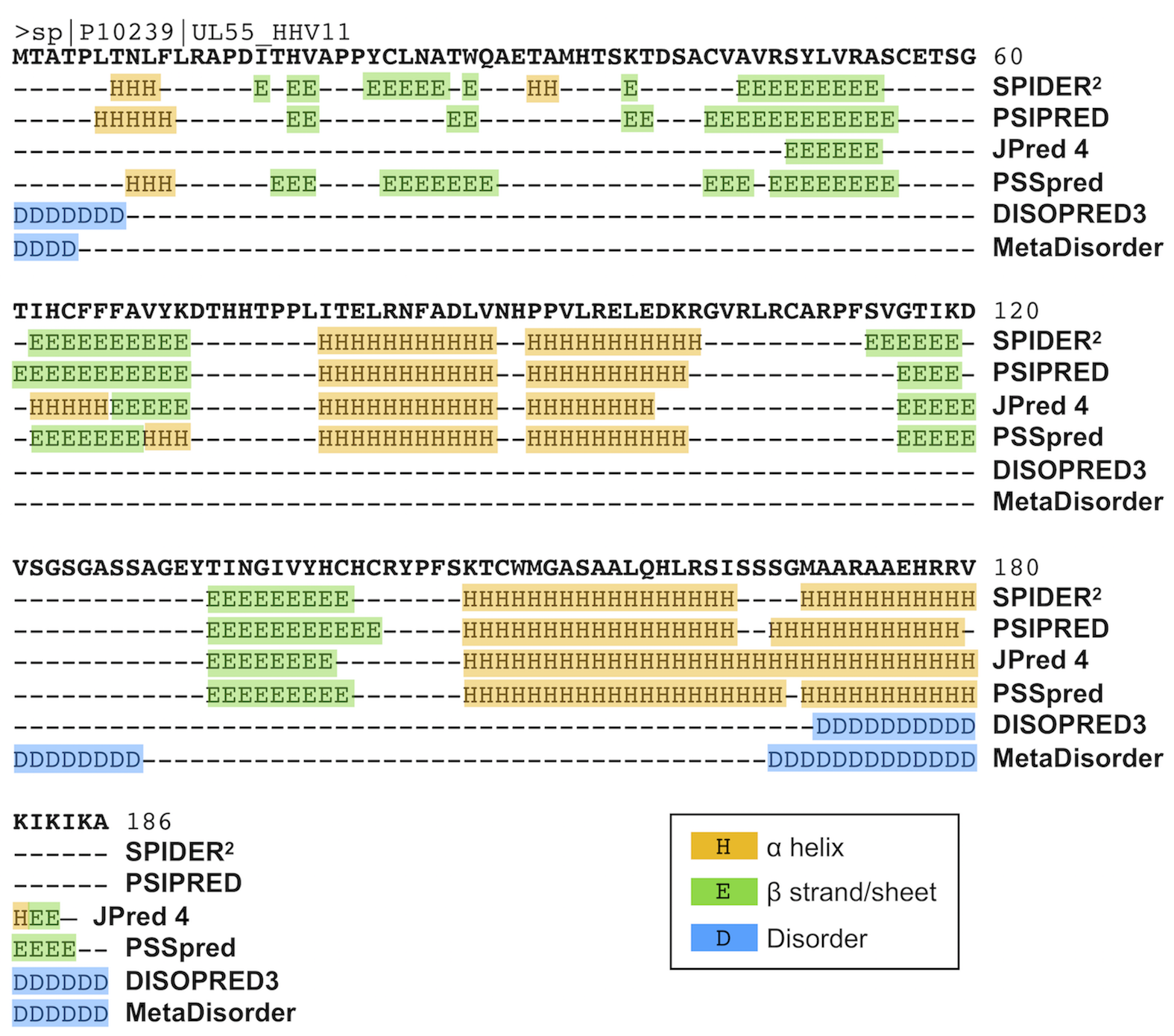

Supplement: S3 Fig — Predictions from each software tool are shown. Predicted disordered regions, α-helices, and β-strands are indicated in blue, yellow, and green, respectively. The final assignment of the secondary structure elements was based on the consensus of individual methods (prediction confidence scores were not taken into account). pUL, protein in unique long region. (TIFF) [file pbio.3000316.s009.tiff]
